# A Decade of Organs-on-a-Chip Emulating Human Physiology at the Microscale: A Critical Status Report on Progress in Toxicology and Pharmacology

**DOI:** 10.3390/mi12050470

**Published:** 2021-04-21

**Authors:** Mario Rothbauer, Barbara E.M. Bachmann, Christoph Eilenberger, Sebastian R.A. Kratz, Sarah Spitz, Gregor Höll, Peter Ertl

**Affiliations:** 1 Faculty of Technical Chemistry, Institute of Applied Synthetic Chemistry and Institute of Chemical Technologies and Analytics, Vienna University of Technology, Getreidemarkt 9/163-164, 1060 Vienna, Austria; barbara.bachmann@tuwien.ac.at (B.E.M.B.); christoph.eilenberger@tuwien.ac.at (C.E.); s.kratz@em.uni-frankfurt.de (S.R.A.K.); sarah.spitz@tuwien.ac.at (S.S.); gregor.hoell@tuwien.ac.at (G.H.); 2 Austrian Cluster for Tissue Regeneration, 1200 Vienna, Austria; 3 Karl Chiari Lab for Orthopaedic Biology, Department of Orthopedics and Trauma Surgery, Medical University of Vienna, Währinger Gürtel 18-22, 1090 Vienna, Austria; 4 Ludwig Boltzmann Institute for Experimental and Clinical Traumatology, Allgemeine Unfallversicherungsanstalt (AUVA) Research Centre, Donaueschingenstraße 13, 1200 Vienna, Austria; 5 Drug Delivery and 3R-Models Group, Buchmann Institute for Molecular Life Sciences & Institute for Pharmaceutical Technology, Goethe University Frankfurt Am Main, 60438 Frankfurt, Germany

**Keywords:** organs-on-a-chip, body-on-a-chip, micro-physiological systems, bioprinting, lung-on-a-chip, liver-on-a-chip, skin-on-a-chip, kidney-on-a-chip, heart-on-a-chip

## Abstract

Organ-on-a-chip technology has the potential to accelerate pharmaceutical drug development, improve the clinical translation of basic research, and provide personalized intervention strategies. In the last decade, big pharma has engaged in many academic research cooperations to develop organ-on-a-chip systems for future drug discoveries. Although most organ-on-a-chip systems present proof-of-concept studies, miniaturized organ systems still need to demonstrate translational relevance and predictive power in clinical and pharmaceutical settings. This review explores whether microfluidic technology succeeded in paving the way for developing physiologically relevant human in vitro models for pharmacology and toxicology in biomedical research within the last decade. Individual organ-on-a-chip systems are discussed, focusing on relevant applications and highlighting their ability to tackle current challenges in pharmacological research.

## 1. Introduction

Over the last three decades, microfluidic systems have evolved from simple chemical sample handling tools to bioanalytical devices and sophisticated cell culture systems. Microfluidic technology builds on microfabrication techniques established within the semiconductor industry to manipulate fluids within micrometer-sized channels. The application of sophisticated microengineering techniques allows careful tailoring of microchannel design, geometry, and topography, leading to precise control over fluid behavior [1]. By translating these engineering techniques from microfluidic chemical reactors to cell-based microfluidic devices, novel biomedical research models can attain unprecedented control over cell behavior in vitro.

Cell-based microfluidic devices recapitulate essential organ functions by mimicking spatiotemporal cell architecture, heterogeneity, and dynamic tissue environments. Consequently, cell-based microfluidic devices are often termed micro-physiological organ-, multi-organ, or human body-on-a-chip systems. In 2016, the world economic forum named organs-on-a-chip models as one of the top 10 emerging technologies due to their prospective economic impact and ability to mimic physiologic organ functions in vitro [2]. The main advantage of integrating complex biology in microfluidic devices is microfluidic systems’ inherent ability to control crucial tissue-specific parameters such as flow rates, temperature profiles, gradients, and biomechanical cues [3,4,5,6,7,8,9]. As a result, several microfluidic cell culture systems have been developed over the years to study the physiologic interplay between organs and tissues as well as the onset and progression of various diseases. The initial concept of recreating tissue and organ communication under controlled measurement conditions was introduced in 2004 by Albert Li and co-workers. They developed the ‘integrated discrete multiple organ culture’ (idMOC) for pharmacological and toxicological drug screenings based on a “well-in-well” principle in a microtiter plate format [10]. The idMOC system benefits from the constant exchange of soluble cues between different cell types, which strongly influences tissue and organ function in vitro. Building on these idMOC systems, Michael Shuler’s and colleagues at Cornell University developed multi-compartment cell culture systems incorporating acellular microfluidic channels to control flow rates, shear, load, and strain forces to simulate the human vasculature. These ‘Micro Cell Culture Analogues’ enabled, for the first time, indirect cell-to-cell communication via molecule exchange between individual organ compartments [11].

Nowadays, referred to as ‘Human-on-a-Chip’ and ‘Body-on-a-Chip’ systems, they incorporate a functioning circulatory system to supply tissue cultures with nutrients and drugs while simultaneously controlling fluid resident time within multiple organ cultures by adjusting the geometries of the micromachined culture compartments. Building on this pioneering work, next-generation organ-on-a-chip systems have integrated microvalves and micropumps, pioneered by Mathies, Maeda, and Quake groups, to precisely control mixing, shear force, and fluid velocities within the microfluidic channel network [12,13,14].

Organ-on-a-chip systems emulate organ functions by incorporating multiple cell types within a three-dimensional (3D) tissue microenvironment [15,16]. In addition to simulating the native cellular microenvironment by applying microfluidic engineering techniques, cell-based lab-on-chip systems allow the incorporation of biosensing solutions to enable on-chip analysis as an alternative to strictly endpoint-based analysis routinely employed in biomedical research [17]. As a result, organ-on-a-chip systems can reproducibly monitor human physiological and pathological processes via optical and electrical sensing strategies to identify dynamic cellular behavior with high structural resolution [18]. Organ-on-a-chip system design centers on three key parameters to mimic a physiologically relevant micro-niche: (1) the specific native tissue architecture, (2) incorporating dynamical stimulation, and (3) emulating spatiotemporal biochemical concentrations [19]. The precise control over geometrical characteristics allows arranging tissue cells according to nature’s blueprint and has led to the establishment of functional vasculature [8,20], or placental [21,22] and blood-brain barriers [23]. Furthermore, the human body’s dynamic mechanical environment is recreated by repurposing pneumatic actuation principles originally designed to move fluid via the deformation of flexible membranes. In organ-on-a-chip systems, flexible membranes stretch cell layers to simulate rhythmic contraction or inflation in muscle [24,25,26], heart [27], lungs [28], and bone tissue [29]. Novel micro-physiological organ systems enable biomedical researchers to understand physiological organ-level functions, investigate deregulation during pathogenesis, and explore treatment and regeneration options [30].

Organ-on-a-chip systems can potentially improve target discovery and toxicity screening in drug development while also offering an opportunity to refine, reduce, and replace animal testing in preclinical trials [31]. The replacement of animal models in biomedical research not only resolves ethical issues associated with animal use but could also accelerate drug discovery while reducing costs (please see other comprehensive reviews for more details) [32,33,34]. In turn, accelerated drug pipelines offer the possibility to explore and evaluate new medical concepts, such as nanomedicine, efficiently [35,36]. As a new paradigm in medicinal nanotechnology, nanomedicine has recently gained attention as therapy in a broad spectrum of diseases [37]. Examples of commonly used materials range from lipids, phospholipids, polymers, and proteins to inorganic materials. Some of them, such as FDA-approved liposomes (e.g., Doxil^®^) or nanoparticles (e.g., Abraxane^®^), are already widely used for the clinical treatment of breast cancer [38]. In these pharmacological and toxicological areas, organ-on-a-chip technology offers a broad spectrum of techniques that can be influential in improving the outcome and predictiveness of in vitro screening technologies. However, the relevance of these microscale technologies for human physiology, disease, and pharmacology first need to be tested. As a consequence, this review summarizes and critically assesses recent advances in the field of organ-on-a-chip technology, including both single-organ as well as multi-organ models with a specific focus directed toward their applications in pharmacology and toxicology.

## 2. Lung-on-a-Chip Systems: Biomechanically Improved Disease Models

**Figure 1:**
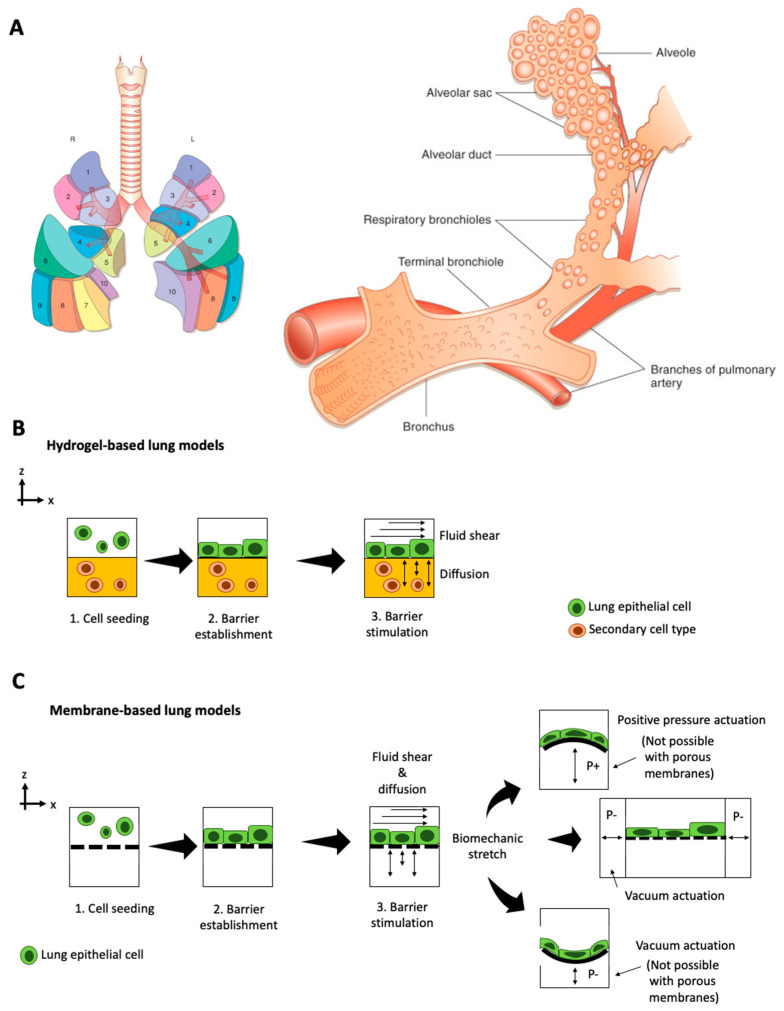
Overview over (**A**) functional lung anatomy (Reproduced from Reference [40] with permissions), as well as biochip approaches including (**B**) hydrogel-based lung-on-a-chip and (**C**) membrane-based lung-on-a-chip with different barrier stimulation and actuation principles. (positive pressure P+; negative pressure P).

The respiratory system consists of a conducting zone (trachea, bronchi, bronchioles) from where inhaled air travels to the respiratory zone (alveolar ducts to alveoli sacs) for gas exchange [39]. Overall, the lung lobules are separated into segments that are individual functional units comprising a bronchus and an artery [40] (see Figure 1A). Pathologies of the lung and respiratory system range from asthma, chronic obstructive pulmonary disease (COPD), bronchitis, and lung cancer to cystic fibrosis [41]. Current preclinical models for human pulmonary diseases are either based on in vivo rodent models that can only mimic certain aspects of human pathologies [42,43], or on ex vivo lung tissue. Ex vivo approaches that use millimeter-thick lung slices of living lung tissue propose an intriguing alternative to current animal models by reflecting the lung’s cellular architecture, structure, function [44], and airway contraction-relaxation dynamics, but these methods are limited by tissue availability [45]. Furthermore, explant cultures can only be tested for a limited experimental time window of days to weeks, which is a major problem for long-term investigation of chronic disease situations. Recent advances in stem cell technology, namely the possibility to induce pluripotent stem cells (iPSC) from somatic cells, allow the recreation of human living lung tissue and associated pathologies in vitro. Using patient-derived progenitor cells (e.g., from cystic fibrosis) [46,47,48,49], complex 3D lung spheroids and organoids can generate mature alveolar epithelium in the lab [50]. These in vitro models offer in vivo-like biological complexity but still lack basic biomechanical cues crucial for lung physiology. In contrast to such static in vitro lung models, lung-on-a-chip models can mimic breathing motions via stretchable membranes to incite epithelial differentiation and physiologic functionality [28]. Microfluidic lung models (shown in Figure 1B,C) incorporate a hydrogel matrix or a porous polymer membrane to mimic the basal membrane in the alveolar epithelium that is subsequently and rhythmically actuated to simulate breathing motions (see overview in Table 1).

The field of micro-physiological models for human lungs, known as ‘lung-on-a-chip systems,’ originated in 2010 when Huh et al. established one of the first biomimetic microfluidic lung models capable of exposing alveolar cells to dynamic stretching [28]. In their device, apical human alveolar epithelial cells, and basolateral microvascular endothelial cells are co-cultured on either side of a porous, flexible poly-dimethylsiloxane (PDMS) membrane to recapitulate an alveolar-capillary interface. By applying vacuum to pneumatic channels on either side of the membrane, the device rhythmically stretches the cell cultures to simulate breathing motions. This physiologic, dynamic environment results in higher magnitudes in transepithelial electrical resistance (TEER) values indicate improved barrier integrity than static culture conditions. The dynamically stimulated cell cultures exhibit in vivo-like physiological responses to *E. coli* nanoparticle exposure and simulate lung edema by reacting to interleukin-2 stimulation with increased barrier leakage. Moreover, the on-chip lung cultures can be used to simulate diseases, such as edema [51]. When adding interleukin 2 (IL-2) to the vascular compartment, the cell barrier started to leak liquid into the alveolar channel. Edema formation was even stronger under cyclic mechanical stretching, which demonstrates the importance of adding add biomechanical loading in a physiological in vitro model. To validate their model, the authors tested the inhibitory effect of transient receptor potential vanilloid 4 (TRPV4) anti-edema drug GSK2193874 to recover barrier integrity, which is similar to drug screening studies in animal models [52]. Additionally, other lung anatomies can be mimicked by varying cell types, as demonstrated in a small airway-on-chip model [53]. In the small airway-on-chip model, the cells respond with asthma and COPD-like phenotypes to interleukin stimulation. The authors demonstrated synergistic effects of lung endothelium and epithelium on cytokine secretion, identified new biomarkers of viral disease exacerbation (M-CSF) that is induced artificially with polyinosinic-polycytidylic acid (poly(I:C), and measured responses to anti-inflammatory compounds that inhibit cytokine-induced recruitment of circulating neutrophils under dynamic culture conditions. In addition to deciphering disease mechanisms in vitro, the small-airway-on-a-chip system can be connected to a device that generates cigarette smoke for alveolar epithelial exposure, leading to ciliary pathologies and COPD-specific molecular signatures [4].The most recent study on this small airway-on-a-chip model by Nawroth et al. [54] looked into rhinovirus-induced asthma exacerbation when infected with live human rhinovirus 16 (HRV16). In this particular study, the chip-based airway model recapitulated viral infection of asthmatic airway epithelium and neutrophil transepithelial migration, which was greatest when viral infection was combined with IL-13 treatment, while treatment with a CXCR2 antagonist reduced neutrophil diapedesis, concluding that IL-13 plays an inhibitory role in immune response modulation against rhinovirus infection. This basic principle can potentially be exploited for drug screening studies on immunomodulatory drugs, such as Janus Kinase (JAK) inhibitors. Furthermore, this micro-engineered airway lung-on-chip example provides a human-relevant model to study the system behind viral-induced asthma exacerbation. The model shows that IL-13 may impair the hosts’ ability to mount an appropriate and coordinated immune response to rhinovirus infection [55].

Another lung-on-a-chip system based on flexible porous PDMS membranes was established by Stucki et al., who integrated a bio-inspired respiration mechanism to investigate the mechanical stretching effects on the air-blood barrier within the lung [56]. In contrast to the lung-on-a-chip systems developed by Huh et al. that stretch the cells parallel to the cell layer, this device mimics diaphragm motions by deflecting a circular membrane perpendicular to the cell surface. However, while integrating a physiologic 3D breathing motion is intriguing, varieties in presented data and cell-type hamper a direct comparison of the devices and cellular reaction. These difficulties in comparing lung-on-chip cultures demonstrate that novel devices need to integrate in-line sensing methods to monitor breathing motions and assess barrier integrity as an epithelial functionality indicator. For example, Mermoud et al. integrate a PCB-based micro-impedance tomography sensor that accurately monitors membrane deflection and tracks lung barrier integrity [57]. This technology significantly improves in-line monitoring of cell barrier integrity compared to conventional tetra-polar TEER measurements routinely employed in other lung-on-a-chip systems [58,59,60].

In addition to the epithelial layer’s mechanical actuation, fluid perfusion and shear stress are essential biomechanical parameters for lung physiology. Lung-on-a-chip devices emulate blood flow within alveolar capillaries by perfusing fluid through endothelial-cell lined channels. This type of fluid perfusion significantly enhances physiologic endothelial cell responses and increases intracellular surfactant-containing laminar body formation and surfactant secretion in type II alveolar epithelial cells [61]. In addition to enhancing physiologic cellular phenotypes, microfluidic device perfusion can be used to perfuse whole blood and investigate blood clot formation in pulmonary embolism [62]. Pulmonary thrombus formation within a primary alveolus-on-a-chip system is stimulated by lipopolysaccharides and endotoxins, while a prospective antithrombotic therapeutic agent to treat pulmonary thrombosis prevents blood clotting. Reconstituting the dynamic microenvironment of the blood vessel-tissue interface via perfusion is imperative for whole blood perfusion and investigation of circulatory cell migration into the alveolar epithelium during inflammation under physiologic conditions [63]. To enable the use as a dynamic, perfused Transwell system, lung-on-a-chip systems need to include porous membranes for co-culturing epithelial and endothelial cells. The integration of flexible and biocompatible alternatives to PDMS as culture substrates is inherently challenging but needs to be achieved to study drug responses reliably. For example, Yang et al. introduced an electrospun poly(lactic-*co*-glycolic acid) nanofiber membrane in their lung-on-a-chip microdevice to co-culture airway epithelial and endothelial cells for drug screening [64] and Humayun et al. suspended hydrogels as a scaffold to spatially align different cell types within a microfluidic lung airway-on-a-chip [65]. The main advantage of using a physiological relevant hydrogel membrane is recapitulating the interface of airway epithelial cells and airway smooth muscle cells. Furthermore, Zuchowska et al. established a less frequently used culture technique with their 3D lung-on-a-chip model based on cancer and healthy lung spheroids to evaluate the anti-cancer activity of photodynamic therapy procedures using prodrug 5-aminolevulinic acid [66]. Overall, lung-on-a-chip devices succeed in mimicking the dynamic lung environment by including physiologic stretching motions and vascular perfusion and reward physiologic cellular behavior as a result. However, the use of these models in preclinical drug development demands the introduction of in-line sensing strategies and the replacement of hydrophobic molecule-adsorbing PDMS to permit viable medium-throughput and high-throughput candidate screening.

## 3. Skin-on-a-Chip Systems: Combined Epidermis Full Skin Thickness Models

As the largest organ of the integumentary system, the skin, built up of several layers of ectodermal tissue, provides the first line of defense against potentially harmful external factors. While ex vivo models of the skin provide a well-established platform for substance testing due to their intrinsic ability to present the hierarchical organization of this barrier, they are limited by both tissue availability and short-term viability. In an effort to overcome these limitations, a variety of companies, including Episkin (Lyon, France), MatTek (Ashland, MA, USA), CellSystems (Troisdorf, Germany), Henkel AG (Dusseldorf, Germany), StratiCELL (Gembloux, Belgium), J-TEC (Aichi, Japan), and Biosolution KO (Seoul, Korea) have invested in the development of commercial in vitro epidermis as well as full-skin models, primarily based on the traditional Transwell set-ups. In addition, significant progress has been made in developing microfluidic skin-on-a-chip models by improving the static Transwell assembly (see overview in Table 2). For instance, Alexander et al. developed a skin-on-a-chip device capable of monitoring both TEER and extracellular acidification rates in an air-liquid interface model [67]. Using this device, the authors were able to detect the impact of sodium dodecyl sulfate in a co-culture of murine fibroblasts (L929) and commercial EpiDerm™ RhE 3D keratinocyte-derived 3D tissues. In their upscaled approach, Sriram et al. developed a mass-producible four-layer platform based on the polymer polymethyl methacrylate (PMMA) to establish skin equivalents with minimal hydrogel shrinkage [68]. After the dermal equivalent based on PEG-fibrin-embedded fibroblasts was established, at day 4, a co-culture with *N*/TERT-1 keratinocytes were initiated at the apical side before switching the culture to air-liquid-interface cultivation. While TEER measurements confirmed improved barrier integrity, immunohistochemical analysis revealed a more pronounced emulation of the epidermal tissue architecture when compared to static Transwell systems. In order to model the epidermal and dermal tissue more accurately, Mori et al. developed a skin-on-a-chip system containing perfusable vascular channels as well as an air-liquid-interface [69]. To that end, endothelial cells (HUVECs) were seeded into a microchannel formed within a fibroblast (NHDFs) embedding collagen hydrogel. After seeding normal human epidermal keratinocytes (NHEKs) on top of the hydrogel construct to create a skin-air-interface in the second step, the uptake of the compound isosorbide dinitrate and caffeine were evaluated. By employing a perfused microfluidic model, Lee at al. demonstrated the importance of fluid flow on the viability of a vascularized 3D skin model exhibiting dermal and epidermal features [70]. Moreover, using a skin-on-a-chip platform, Alberti et al. could confirm similar diffusion behaviors of caffeine, salicylic acid, and testosterone upon comparison to an in vitro skin permeation assay based on a static Franz diffusion cell loaded with similar organotypic skin equivalents while reducing sample variation by half [71].

To facilitate the handling of perfused microfluidic devices, Song et al. opted for the development of a pumpless passive flow device to co-culture fibroblasts and HaCaT keratinocytes. While the model was validated by assessing matrix remodeling via the expression of the extracellular matrix (ECM) components Collagen IV, Keratin X, and fibronectin, the study could also link collagen sources to keratinocyte differentiation capacity [72]. In their passively driven device, Abaci et al. established an air-skin-interface model for the monitoring of cutaneous drug uptake [73]. Herein, the authors revealed the direct toxic effects mediated by the chemotherapeutic drug doxorubicin on keratinocyte proliferation and differentiation.

Wufuer et al. induced a TNF-α-mediated edema in their skin-on-a-chip model to evaluate dexamethasone as a treatment option for the swelling of both the epidermis and dermis [74]. Consisting of three stacked compartments with each separated by a porous PET membrane, this device comprises an epidermal, dermal, and vascular layer to assess drug translocation. An improved micro-physiological immune-competent HaCaT keratinocyte-based skin model capable of modeling human skin allergy responses was introduced by Ramadan et al. who employed integrated TEER measurements to assess the impact of the immune system on the barrier integrity of the skin construct following exposure to lipopolysaccharides as well as UV light [75]. Environmental factors, such as UVB irradiation, are particularly interesting because skin irradiation is believed to induce cutaneous barrier deterioration in skin xenografts as well as skin equivalent models by downregulating tight junctions (claudin-4) [76]. Another technological leap forward includes the integration of the hypodermis featuring white adipose tissue since adipose tissue residing beneath the dermis can act as an uptake reservoir for hydrophobic pharmacologically substances [77]. Loskill et al. investigated the properties of white adipose tissue in a micro-physiological system using preadipocytes differentiated on a chip [78]. This adipose-tissue-on-a-chip allowed for the incorporation of features specific to adipose tissue, such as collagen secretion and lipid droplet formation. Only the on-chip combination of the epidermis with vascularized full thickness skin, including adipose tissue, will be able to provide a translatable and physiologically relevant alternative model for pharmaceutical and pharmacokinetic uptake studies. Furthermore, future on-chip skin models will need to account for the immune system as well as the nervous system to create a miniaturized model that resembles physiological skin [79]. From an anatomical viewpoint, skin-on-a-chip systems also need to consider skin appendages, i.e., follicles and glands, which can be grown with organoid technology [80], as demonstrated by Lee et al. These structures are vital for control of temperature, fluids, and external stress. Furthermore, similar to the lung-on-a-chip strategy, biomechanical cyclic strain should also be considered for future models as loading is vital for skin homeostasis while overuse can stimulate fibrosis pathways [81,82].

## 4. Liver-on-a-Chip: Valid Models for Pharmaceutical Screening

**Figure 2:**
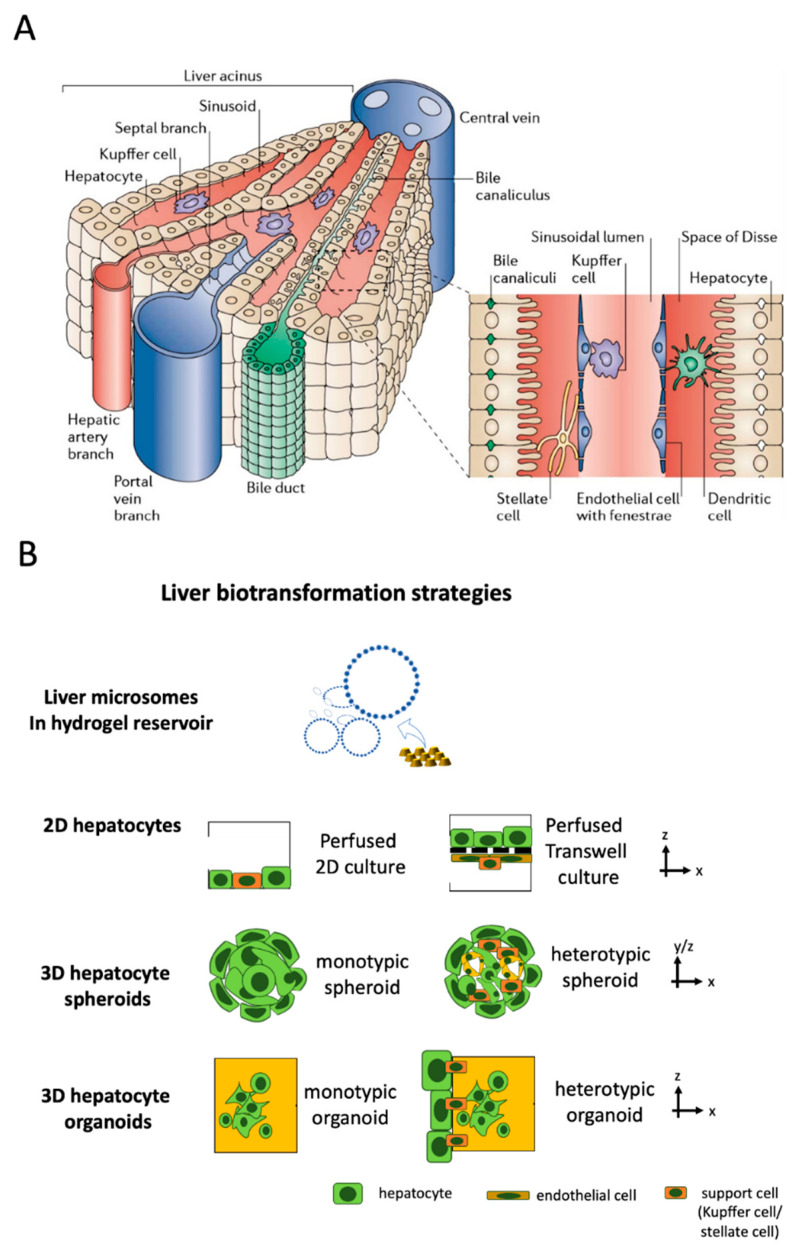
(**A**) Structure of liver acini and sinusoids. (Reproduced from Reference [83] with permissions of Elsevier 2021). (**B**) Biotransformation strategies on-chip mimicking liver metabolism using acellular microsome or hepatocyte-based approaches.

The liver is the largest internal human organ and is involved in drug metabolism (first pass effect) and detoxification as well as other metabolic functions that regulate blood glucose levels, bile synthesis, and the production of various plasma proteins, such as albumin. Anatomically, the liver is built up by liver acini, which are the functional units of the liver comprising liver sinusoids that are connected to a complex network system of hepatic artery, portal vein, central vein, and bile ducts [83] (see Figure 2A). Due to its overall importance in drug metabolism, the liver is a major focus of pharmaceutical research [84]. The liver, as a vital organ, plays an essential role in protein synthesis and xenobiotic metabolism and exhibits a high degree of regenerative capacity. However, drugs, toxins, viral infections, cancer, and even traumatic injuries can still result in permanent tissue damage and liver function impairment, which will eventually lead to end-stage liver disease or acute liver failure [85,86]. To date, human liver slices as well as rodent animal models have been widely used for drug screening and toxicity testing in pharmaceutical research as well as models for pathophysiological studies on non-alcoholic- and alcoholic fatty liver disease (NAFLD/ALD) [87,88,89,90].

As a result of the limited availability of human tissue samples and the ambiguous findings from animal models that often do not translate well to humans [91], a number of chip-based cellular and acellular liver models using a minimal number of human cells at minimal tissue-like architectural features have been developed in recent years, as outlined in Figure 2 (see overview in Table 3). The four basic approaches depicted in Figure 2B can be found to recapitulate liver-function using liver-on-a-chip systems for a range of organotypic complexities. To further improve biomimetic architecture and organotypic function, hepatocytes can be co-cultured with endothelial and other support cells (i.e., Kupffer or stellate cells) as 2D monolayers on microchannels and membranes, multi-layered 2.5 D co-cultures on membranes and interfaces as well as multi-cellular spheroids (MCS) and heterotypic hydrogel-embedded multi-co-cultures, respectively, to recapitulate various degrees of cellular functions and tissue architectures found in vivo in liver sinusoids (see Figure 2B).

A common and important physiological parameter of in vitro liver systems for pharmacological studies is defined by the metabolic cytochrome P450 activity of hepatocytes [92]. For instance, reaction kinetics of P450 enzymes encapsulated within liver microsomes generated from photosensitive of polyethylene glycol diacrylate (PEG-DA) gels were used by Lee et al. to simulate the liver function without the use of hepatocytes [93]. This study demonstrated that acellular substitutes can be employed as a simple model of the liver metabolism for pharmaceutical research on a chip. However, two-dimensional (2D) hepatocyte monolayers and 3D liver constructs were widely used as in vitro liver models for drug screening applications and disease mechanism studies. An important premise of any liver model for drug screening and pathophysiological studies is the establishment of a well-organized liver microtissue that recapitulates both the structural, physiological features and liver-specific functions of the native liver through the co-culture with non-parenchymal cells and the re-creation of the liver ultrastructure, using micromachining techniques [94]. The key aspect of liver-on-a-chip technologies is the implementation of dynamic fluidic flow conditions to generate a biomechanical, organotypic culture microenvironment. A number of studies based on monolayer cultures of rat and human hepatocytes as well as those using 3D liver spheroids based on HepG2 and iPSC-derived hepatocytes have shown that the presence of fluid flow ensures reliable delivery of important soluble factors in a controlled manner, resulting in improved viability and enzymatic activities (e.g., CYP1A1, CYP1A2 and CYP3A4) [95,96]. For instance, using a three-dimensional liver-on-a-chip system, Lee et al. demonstrated the importance of supporting hepatic stellate cells (HSCs) to maintain hepatocyte spheroids, especially the formation of tight cell–cell contacts, thereby, significantly improving liver-specific function and resulting in enhancing cytochrome P450 activity [97]. Another important application of hepatic models includes drug screening and chemical-safety testing. These approaches require functional matured hepatocytes and human pluripotent stem cell (hPSCs) technology that is considered one alternative to provide robust and highly pure hepatic clusters. For instance, Kamei et al. developed a liver-on-a-chip platform using mature and functional hepatocyte-like cells derived from hPSCs [98], to create a 3D culture environment in which hepatocyte-like cells exhibited increased expression of hepatic maturation markers and cytochrome P450. The improved cultivation conditions led to a significantly elevated drug uptake/excretion capabilities above 90%, thus, highlighting the benefits of using human iPSC-derived hepatocytes as a substitute for immortalized as well as primary hepatocytes in drug screening and chemical safety testing. To model alcoholic liver disease, Deng et al. simulated liver sinusoid function by co-culturing HepG2 hepatocytes with LX-2 stellate cells, EAhy926 endothelial cells, and U937-derived Kupffer cells in a multi-layered double-membrane liver-on-a-chip system that displayed improved viability and increased urea and albumin levels [99]. The authors also demonstrated that exposure to alcohol damages the tight junctions of hepatocytes, reduces the release of NO of endothelial cells, and stimulates stellate cell proliferation. A similar study confirmed the validity of this complex on-chip co-culture model by using human primary hepatocytes instead of cell lines [100]. Another example of the on-chip co-culture system includes primary human hepatocytes and liver sinusoidal endothelial cells (LSECs) with stellate and Kupffer cells. Here, Li et al. investigated the role of zonation in hepatic physiology, hepatotoxicity, and progression of liver diseases using their liver acinus model. The endothelial compartment lined with human LSECs recapitulated immunologic functions within the liver sinusoid and promoted polymorphonuclear leukocytes’ adhesion and transmigration upon activation. Furthermore, zonation-specific differential oxygen gradients lowered hepatic mitochondrial activity and hepatic steatosis but triggered increased α-SMA expression and proliferation of stellate cells at 4% oxygen [101]. Another sinusoid-on-a-chip system was developed by Kang et al. introducing a less complex, animal-derived liver model to analyze viral replication for hepatitis B virus (HBV) [102].

In a different study, a liver-resembling microfluidic device called ‘Exoliver culture’ showed not only the main characteristics of the liver sinusoid including cellular architecture, biodynamic angiocrine stimulation, and parenchymal zonation, but also responded differently to acute treatment with known hepatotoxic drugs than those seen in conventional culture platforms [103]. An acute toxic insult of 100 µM troglitazone did not show any effect on the Exoliver platform even though IC_50_ values for troglitazone toxicitiy is around 10 µM [104]. Considering that the clinical c_max_ values [105] for drug-induced liver injury (DILI) of troglitazone are around 5 µM, the Exoliver platform shows low sensitivity with lack of increase of soluble keratin 18 levels compared to spheroidal human liver models that are more sensitive for drug assessment in vitro (IC_50_ 5–10 µM). Furthermore, even though a dual species approach was initially investigated for the liver phenotype, the drug screening efforts were performed only for human liver biochips. However, species-related differences are very strong when comparing the liver toxicity model, i.e., of human and rat origin. In a cross-species study comparing humans with rat and dog models, Jang et al. [106] investigated organotypic liver function as a drug screening and disease modelling platform to investigate species-specific responses. For instance, a human liver-on-a-chip could predict dose-dependent lipid accumulation, albumin reduction, and release of liver injury markers as an indicator for steatosis caused by fialuridine (FIAU). Neither a rat liver-on-a-chip nor a rat animal model could have predicted species differences in steatosis because such disease mechanisms require human or humanized models. Furthermore, this study shows that liver-on-a-chip technology should be considered complimentary technology to investigate other aspects of toxicology including the formation and effect of reactive metabolites in DILI at different architectural levels (i.e., cell as well as tissue context) to fill the knowledge gap between dynamic tissue responses and clinical patient pathologies. In addition, using the proposed cross-species approach, a basic pre-screening can identify a reactive and predictive system for more in-depth in vivo tests on a case-to-case basis (e.g., wildtype or humanized rat strain, etc.).

Even though liver-on-a-chip platforms are organotypic models to recapitulate liver functions using various cell types and co-culture techniques for basic science, these biomimetic systems still lack scalability for high-throughput experimental setups as the biochips only feature one or two individual liver units per chip. Combining protocols for generating liver-on-a-chip systems with more high-throughput technologies, such as the Mimetas OrganoPlate^®^ or other microtiter formats, would increase the relevance of biomimetic liver modeling in vitro for drug and toxicity screening, which is clearly one major challenge for a broad application of such liver microsystems. Since pre-screening as well as high-content analysis do not require extensive throughput, liver-on-a-chip technology should be seen as a valuable ‘gap filler’ to improve the molecular understanding of the tissue response prior to animal models and clinical trials. The particular impact of how liver compartments change the pharmacological and metabolic predictiveness of multi-organ-on-a-chip or body-on-a-chip systems will be reviewed later on Section 10. Multi-organ on-chip models show combined tissue models to screen drug-drug interactions, pharmacokinetics, and pharmacodynamics.

## 5. Kidney-on-a-Chip: Evaluating Nephrotoxicity and Drug Efficacy

The kidney plays a major role in maintaining homeostasis of the internal circulation. It regulates blood pressure, maintains a physiological pH, retains essential nutrients, and eliminates waste molecules, including drugs and drug metabolites. To this end, the kidney receives a large cardiac output volume, has a high metabolic turnover rate, shows extended concentrating abilities, and is capable of efficiently trafficking compounds, which may increase sensitivity to drug-induced adverse effects at the kidney. Acute kidney injury is observed in a clinical setting, where 20% of all acute kidney injury cases are caused by kidney toxic drugs [107], strongly suggesting that current drug toxicity evaluation procedures need to be improved [108]. Kidney dysfunction resulting from drug side effects remains an important issue to consider in the drug development process. It is important to point out that traditional in vivo animal experiments are particularly limited with respect to evaluating drug efficacy and nephrotoxicity because of the apparent discrepancies in drug pharmacokinetics and pharmacodynamics between humans and animals. Recently, several types of human kidney-on-a-chip systems have been developed that reflect the microenvironment of the kidney tubule and have shown similarity to in vivo results of drug nephrotoxicity. Using kidney-on-a-chip systems, investigators can measure and evaluate various drug-induced biological responses [109], thus, showing potential advances in disease modeling and drug lead optimization studies (see overview in Table 4). Some bioengineering challenges, however, remain and are mainly concerned with reconstructing the complex architecture, cellular make-up, and matrix composition necessary to model kidney function at an organotypic level. This highly complex process starts at the proper exchange rate during glomerular filtration, followed by tubular secretion and reabsorption, which are all critical in human electrolyte regulation, toxin and metabolite excretion, and acid/base homeostasis [110]. Any alterations of these processes can result in a high incidence of tubular exposure to concentrated drugs and toxins, leading to kidney injury. In terms of structural complexity, the renal tubular system consists of three major structural components: the tubular lumen, the vascular lumen, and a thin layer of a basement membrane separating the luminal spaces, surrounded by the ECM. Furthermore, it is technically challenging to re-engineer the close alignment of vascular and tubular compartments necessary to model solute transfer across the renal tubular exchange interface where three major resident cell types, fenestrated endothelial cells, specialized epithelial cells, and interstitial perivascular cells are present [111]. To address these bio-engineering challenges, Rayner et al. created a tunable human renal vascular–tubular unit formed entirely in collagen hydrogel [112]. Vascular and tubular lumens were assembled against a solute-permeable and mechanically robust collagen membrane and seeded with human kidney microvascular or epithelial cells. These cells gradually remodeled and condensed the collagen membrane to a few micrometers thickness, resembling a native basement membrane, while still maintaining sufficient integrity to support blood perfusion through the vascular lumen. Additionally, kidney-specific function was demonstrated including selective reabsorption of albumin and glucose.

Other kidney-on-a-chip devices incorporate organoids under flow using millifluidic systems to expand the endogenous pool of endothelial progenitor cells and to generate vascular networks with perfusable lumens surrounded by mural cells [113]. Vascularized kidney organoids cultured under flow exhibited, to a greater extent, mature podocyte and tubular compartments with enhanced cellular polarity and adult gene expression compared to static controls. The ability to induce substantial vascularization and morphological maturation of kidney organoids in vitro under flow opens new avenues for studies of kidney development, disease, and regeneration. An aspect that is commonly overlooked is that chemical compounds are also excreted through the kidney and/or biliary tract, thus, influencing drug concentrations within the body. To account for this effect, Kim et al. reported external fluid pumping mechanisms to recreate two types of pharmacokinetic profiles corresponding to either bolus injection or continuous infusion [114]. Nephrotoxicity testing of the antibiotic gentamicin revealed that continuous infusion reduced nephrotoxicity, and prolonged exposure to gentamicin disrupts cell–cell junctions, increased membrane permeability, and decreased cell viability. These results underline the benefits of using microfluidic cell culture models for pharmacokinetics and toxicity studies. In another nephrotoxicity study, a microtiter-plate-based microfluidic 3D proximal tubule on-a-chip was established with renal proximal tubular epithelial cells grown as tubules against an ECM. Exposure to the nephrotoxic, anti-cancer drug cisplatin showed a dose-dependent disruption of the epithelial barrier, a decrease in viability, an increase in effluent lactate dehydrogenase (LDH) activity, and changes in expression of tight-junction markers and inhibition of the crucial drug efflux pumps as *p*-glycoprotein (*p*-gp) and multi-drug resistance protein (MRP) [115].

The glomerulus plays a central role in regulating the filtration of molecules across the renal barrier, whereas small molecules and water pass through the barrier. Conventional kidney systems have a limited ability to evaluate glomerular function, making them unsuitable for nephropathy studies on exposure to various environmental toxins. Li et al. established a kidney-on-a-chip model that facilitates cadmium-induced renal toxicity assessment in a physiologically relevant manner [116]. The toxic metal cadmium, which is a common environmental pollutant, poses a significant health risk to humans and, consequently, a major public health concern. As a representative study of the barrier function, its permeability to different molecules was characterized and quantified. The data support the appearance of pathological changes that are present in glomerular diseases in vivo under cadmium exposure, such as proteinuria. The culture and collection channels represent the capillary and the glomerular capsule sides of the glomerular filtration barrier, respectively. Isolated primary rat glomerular endothelial cells were cultured on the side surface of the middle gel channel to model the selective permeability of the renal barrier. Cadmium induced significant cytotoxicity and disrupted the expression of tight junction protein ZO-1 in a dose-dependent manner and increased the permeability of the endothelial layer to large molecules known as immunoglobulin G and albumin. These results facilitate the understanding of the underlying mechanism of kidney dysfunction and glomerular disease. Huang et al. showed that co-culture of adipose-derived stem cells (ASCs/AdMSCs) as well as kidney epithelial cells impact barrier height and also tight junction formation, resembling a condition closer to in vivo kidney tissue [117].

## 6. Heart-on-a-Chip: Identifying Beneficial and Adverse Effects of Drugs in Health and Disease

The heart’s biomechanical environment, structural complexity, and exposure to dynamic stimuli play a critical role in maintaining cardiac physiological functions. Microfluidic in vitro platforms offer the opportunity to precisely control each of these factors, enabling us to study cardiac physiology and a range of pathologies such as myocardial infarction, arrhythmia, or cardiomyopathy [118] (see overview in Table 5). However, due to the high morbidity of heart diseases and common adverse effects of medication, the heart is an important target tissue for any drug development and interaction study. In other words, more predictive cardiac models will not only foster the identification of novel therapeutic strategies, but also help to screen off-target cardiotoxic adverse effects [119,120]. For instance, Ellis et al. presented a perfused myocardium-on-chip system consisting of spatially controlled co-cultures of human hiPSCs derived myocardial cells (iCMs) and hiPSC derived endothelial cells (iECs) from the same patient, thus, paving the way for personalized medicine [121]. This system enabled the reproduction of microvasculature (with iECs) and cardiac muscle (with iECs and iCMs) of the human myocardium. To assess the effect of ECM on the cardiotoxicity of isoproterenol on cardiomyocytes, Tomecka et al. performed a comparative analysis between (i) 2D-monolayer cultures and (ii) 3D-hydrogel models on a single chip platform [122]. Precise spatial orientation within a scaffold was accomplished by Zhang et al. who utilized 3D bioprinting to create a vascularized myocardium on-chip. Here, human umbilical vein endothelial cells (HUVECs) were printed into a scaffold and rat cardiomyocytes seeded on top to form a myocardium, which showed synchronous and spontaneous beating patterns. Investigation of a cardiotoxic substance (doxorubicin) was conducted by combining the device with a perfusion bioreactor [123]. Aside from the choice of cell types, which are the culture method and architectural considerations described above, electrical and mechanical stimulation constitutes another key parameter in establishing functional cardiac models [124,125]. As an example, Marsano et al. developed an organ-on-a-chip system with improved cardiac cell differentiation after applying mechanical stimuli (e.g., uniaxial cyclic strain) via pneumatic actuation on 3D cell-laden hydrogels confined by hanging posts. The stimulated functional micro-engineered cardiac tissues (μECTs) displayed superior differentiation and better cell-to-cell coupling, which were analyzed electrically and mechanically. The devices’ potential for drug testing applications was demonstrated using isoprenaline, which were 100-times lower concentration in the actuated microtissues (e.g., 1 nM), showed similar toxic responses that are otherwise seen at 100 nM using embryonic cells [126]. Moreover, Chan et al. combined myocytes and cardiac fibroblasts with transfected optogenetic embryoid kidney cells expressing channelrhodopsin-2, channelrhodopsin-16, and channelrhodopsin-17 that activates a light gated ion channel to forward the optical signal to the cardiac cells. Consequently, the muscle strip can be activated by blue light illumination similar to a pacemaker. This non-invasive strategy is able to activate and modulate muscle movements by blue light and it replaces mechanical or electrical stimulation of muscle cells [25]. To increase throughput, Hansen et al. established a multiplexed device capable of performing 24 heart-on-a-chip experiments in parallel using neonatal rat heart cells. A commercially available well plate combined with PDMS pillars allowed the measurement of beating rates and optical analysis of pillar deflection. The drug screening potential of the device was demonstrated by detecting increased relaxation time after the addition of clinical-relevant blockers/drugs (chromanol, quinidine, erythromycin), whereas doxorubicin led to a decrease in the contraction force [127].

Monitoring heart functions using on-chip and off-chip sensing strategies constitutes a major advantage of organ-on-a-chip technology over standard in vitro systems. For instance, Ahn et al. showed the formation and analysis of cardiac tissues grown around muscle-inspired nanofibers using cantilevers. The movement of the cantilevers could be correlated to the contractility of the tissues to investigate effects of TiO_2_ and Ag nanoparticles [128]. A reusable device presented by Agarwal et al. is based on muscular, thin film technology coupled to a microfluidic channel for high throughput drug testing. Cardiac cells were seeded on one side of PDMS cantilevers and contraction was monitored, providing information about contractile responses. Here, microfluidic channels act as inlets/outlets for drugs, as shown with isoproterenol, allowing evaluation of dose-response relationships on cardiac cells using an autoclavable and cost-efficient testing system [129]. Using a multi-material 3D printing approach, a micro-physiological device was fabricated by Lind et al. to monitor cardiac activity based on a continuous electronic readout of contractile stress using multiple laminar cardiac micro-tissues derived from neonatal rat ventricular myocytes (NRVMs) and human-induced pluripotent stem cell-derived cardiomyocytes (hiPS-CMs). By using multilayered cantilevers including a base layer, embedded strain sensors, and a tissue guiding layer, it became possible to observe cardiac activity (contractile stress) of self-assembled micro physio-mimetic laminar tissues [130]. The system was validated by measuring the increased cardiac activity when isoproterenol was administered to the microsystem. Next, Stancescu et al. showed a combination of electrical and contractile activity measurements, where human stem cell-derived cardiomyocytes were cultivated as a 2D monolayer and a multi-electrode array allowed analysis of the beat rate, conduction velocity, and action potential length, whereas cantilevers allowed the contractile force analysis. Key drugs used in clinical studies (sotalol, norepinephrine, and verapamil) were tested with the device and the outcomes were in agreement with clinical data [131]. Similar to microelectrode arrays (MEAs), high speed impedance measurements of cardiac tissues provide information about their mechanical contraction status. Zhang et al. presented a platform with quartz substrate and gold electrodes, which acquires the impedance data and can be used to investigate cardiotoxicity and drug efficacy of the drugs verapamil and doxorubicin [132]. This generation of heart-on-a-chip systems was followed by multi-tissue and multi-organ models that included vascular barriers as well as bystander tissues, such as the liver, due to its high metabolic conversion activity of pro-drugs and pro-toxins to toxic metabolites.

## 7. Vasculature-on-a-Chip: Vascular Barrier Transport and Anti-Vasculogenic Drug Screening

**Figure 3:**
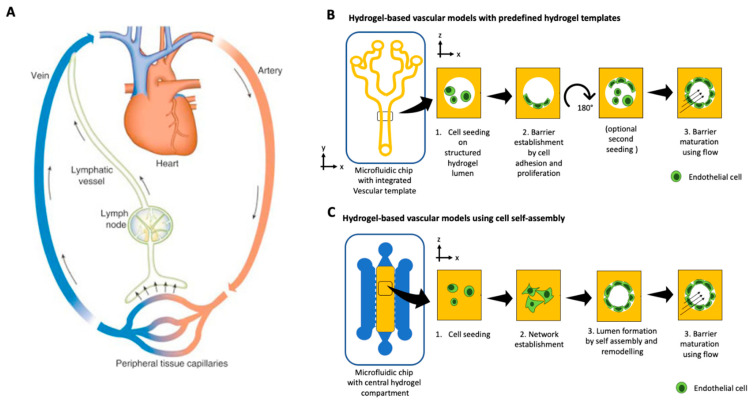
(**A**) Schematic of the circular and closed blood vascular and open-ended lymphatic system. (Reproduced from Reference [145] with permissions from Elsevier 2021.) Hydrogel-based organ-on-a-chip approaches for vasculature using (**B**) a predefined on-chip hydrogel template that is cellularized after printing or (**C**) cellular self-assembly and remodeling of a bulk hydrogel matrix where endothelial cells start to sprout, form a pre-vascular network, and mature over time into a lumenized vascular structure.

Two vascular systems, known as the cardiovascular and the lymphatic system, provide the human body with nutrients and remove waste products. The cardiovascular system transports oxygenated blood, nutrients, and immune cells to every organ and tissue in the body via arteries, arterioles, and a fine interwoven mesh of capillaries. In contrast, the lymphatic system collects excess fluid expelled from the cardiovascular system by shear forces and recirculates it through the lymph nodes for immune control back to the cardiovascular system via passive-pumping motions [133]. Consequently, both vascular systems play an integral part in the absorption, distribution, and excretion of nutrients, drugs, and other compounds [134]. Irrespective of the type of vasculature, vessels are comprised of an inner endothelial cell barrier, controlling cells and molecule passage, and a stabilizing outer layer of pericytes or smooth muscle cells. New vasculature is either formed by de novo vascularization from single progenitor cells or by angiogenesis by sprouting from the existing vasculature [135]. Traditionally, vascular cells have been studied using monolayers in microtiter plates and Transwell cultures or pseudo-capillary structures on hydrogels, simplifying the models to a point where the translation to a human body becomes questionable due to loss of organotypic phenotype and genotype. Organ-on-a-chip technology provides the opportunity to engineer an in vivo-like cellular microenvironment tailored to vascular structures’ needs, including physiological flow, control of direct and indirect cell-cell communication, and ECM-like hydrogel scaffolds (see overview in Table 6) [136,137]. Organ-on-a-chip models for vascular biology can be divided into two approaches, as shown in Figure 3A,B. Firstly, vessel generation by microfluidic-assisted methods, such as for sacrificial molding and, second, by cell self-assembly of vascular structures through vasculo-genesis or angiogenesis from individual cells [138]. Microfluidic-assisted methods for on-chip microvessel generation rely on providing a preformed structural architecture of vessels, either made from hydrogels or the microchannel itself, where endothelial cells are seeded to form an endothelial lining. One method, sacrificial molding, employs a structural placeholder removed before cell seeding to generate open lumina within a hydrogel. Alternatively, the microchannels themselves serve as the vessel structure and are circumferentially seeded with endothelial cells as a vessel replica. As a biomimetic approach, Costa et al. established an in vitro thrombosis model where the authors used angiography data from computed tomography data to create patient-derived 3D-printed vessel constructs of normal and stenotic vessels [139]. These biomimetic biochips were cellularized with HUVEC and perfused with human blood at physiologically shear rates, resulting in induced thrombosis only in pathological geometries. A more frequently used microchannel patterning approach casts a polymer onto molds representing a semi-circular vessel analog and subsequent bonding to another semi-circle to form a fully circular vessel. Some current examples of this method include circumferential alignment of endothelial cells and smooth muscle cells for a model of in vivo-like vasculature [140] or a model for investigation of the effect of topography and substrate rigidity on an endothelial cell layer [141]. Combined with increasingly sophisticated microfabrication techniques [142], microchannel patterning of PDMS on SU8 substrates could be a powerful tool for mimicking in vivo-like capillary structures in the future. As an alternative to PDMS, various hydrogels have been used to produce microchannels. For example, Nie et al. [143] and He et al. [144], recently demonstrated the use of various ECM-mimicking hydrogels and combinations for casting bifurcated networks that represent vessel anatomy.

Furthermore, Menon et al. recently established a novel ECM patterning method for creating perfusable vascularized channels based on the capillary burst valve concept [146]. Using this novel method, the authors achieved various geometries and could mimic neutrophil trans-endothelial migration as well as stenosis in atherosclerosis. The implementation of hydrogel molding techniques in microfluidic devices could go as far as recreating a vessel on chip with the ability of subsequent direct surgical anastomosis [147]. In contrast to hydrogel patterning, another microfluidic assisted approach-sacrificial molding-utilizes a needle-based technique where a hypodermic needle is inserted into a microfluidic structure before injection of a hydrogel [148]. The needle is subsequently removed upon hydrogel polymerization, thus, leaving a cylindrical void in the hydrogel for endothelial cell seeding. The seeded endothelial cells then form an endothelial cell barrier lining the inner walls of the channel, thus, creating a single perfusable vessel. The sacrificial molding method can be employed for: (i) single-cell systems containing only endothelial cells [149], (ii) multicellular systems including supporting cell types such as fibroblasts or stem cells [150], or even (iii) to recreate all three layers (adventitia, tunica media, and tunica negra) of a biomimetic blood vessel [151]. Further examples for successful implementation of the sacrificial molding method include studies to investigate vascular barrier transport [152]. For example, it has recently been employed by Christopher Chen’s group to identify a previously unknown Notch-signaling pathway and elucidate the effect of inflammation on the vascular barrier [153]. Unfortunately, only one model employing sacrificial molding for lymphatic vessels has been reported so far [154]. However, in their model, Gong et al. report a microscale lymphatic vessel (µLYMPH) impressively exhibiting similar permeability and draining functions as in vivo lymphatics. Alternatively, lumenized microfluidic templates with better yield and performance can be created with viscous finger patterning, as demonstrated by Bischel et al. [155], where a lumen inside cell-laden hydrogels can be created prior to polymerization by hydrostatic flow of culture medium. The study of de Graaf et al. [156] investigated the robustness and reproducibility of this technique with different pumping regimes and identified that their extended passive pumping produced the lowest inter-lumen and intra-lumen variations less prone for user variations. The second option for recreating vascular structures in microfluidic devices is the self-assembly of vessels from single cells. Contrary to the microfluidic assisted methods mentioned above, where hollow structures resembling a vessel are generated before cell seeding, self-assembly utilizes vascular cells’ inherent ability to form interconnected vascular structures through angio- and vasculogenesis. The method, pioneered by Roger Kamm’s group, features endothelial cells and supportive cell types resuspended in a hydrogel and, subsequently, loaded into a microfluidic device [157]. Upon device loading, the cells initiate processes similar to vasculogenesis in vivo, resulting in a perfusable vascular network spanning between two medium channels. The endothelial cells and supportive cell types can either be embedded within the same hydrogel for direct cell-cell interaction or separated by a medium channel for vascular assembly via paracrine signaling. These self-assembled microfluidic in vitro vascular networks are an ideal tool for studying basic biologic processes, diseases, and even exposure to drugs or toxic compounds. For example, interstitial flow and growth factor gradients have long been assumed to play a vital role in vasculogenesis. Microfluidic devices can generate and maintain directional interstitial flow and growth factor gradients, demonstrating that both factors play a vital role in endothelial cell sprouting [8,158,159]. The only publication, to date, describing lymphatic endothelial cells in a self-assembly microfluidic setup showed that, while blood endothelial cells preferentially sprout in the direction of the interstitial flow, lymphatic endothelial cells favor angiogenesis toward the interstitial flow, successfully recreating a physiological response emulating the capillary bed [160]. Aside from basic biology, self-assembled vascular structures are routinely employed to study the delivery of drugs or even toxic compounds via the vascular system because on-chip vascular models exhibit similar permeability and transcellular and paracellular transport mechanisms as an in vivo vasculature [161]. As an example, Zeinali et al. investigated the effect of the anti-vasculogenic drug Nintedanib to understand the mechanisms underlying the course of action of routinely administered drugs for idiopathic pulmonary fibrosis [162]. The study found that the drug’s effect was visible even at minimal concentrations, leading to a significant increase of vascular permeability, a decrease of vessel diameter and density, and fibroblast organization around the blood vessels. Finally, self-assembled microfluidic vasculature models have recently been employed to elucidate toxic nanoparticles’ effect on blood vessels. Jeon et al., for instance, report that the oxidative stress upon exposure to cationic polymer nanoparticles leads to a rearrangement of microtubules and vessel contraction before caveolae-mediated transcytosis [163]. Additionally, atmospheric nanoparticles have been found to disturb multiple vascular-associated molecule expressions by forming a protein corona upon inhalation, leading to enhanced vascular permeability by losing tight junctions [164]. Overall, microfluidic models of vasculature have been routinely used to study physiologic and pathologic responses in vitro over the last five years and have proven to be a powerful tool not only for understanding the basic biology involved in vessel formation but also for the effects of drugs and toxic compounds on the human body’s vascular systems. To provide a technology platform that offers better scalability and can be combined with robotic automation, Phan et al. established micro-vasculatures on a 96-well plate format [165] to independently perfuse microvascular organoids by hydrostatic pressure based on their 3D micro-tumor biochip design [166].

## 8. Blood-Brain-Barrier-on-a-Chip: Barrier Function, Transport Properties, and Neuroinflammatory Model

**Figure 4:**
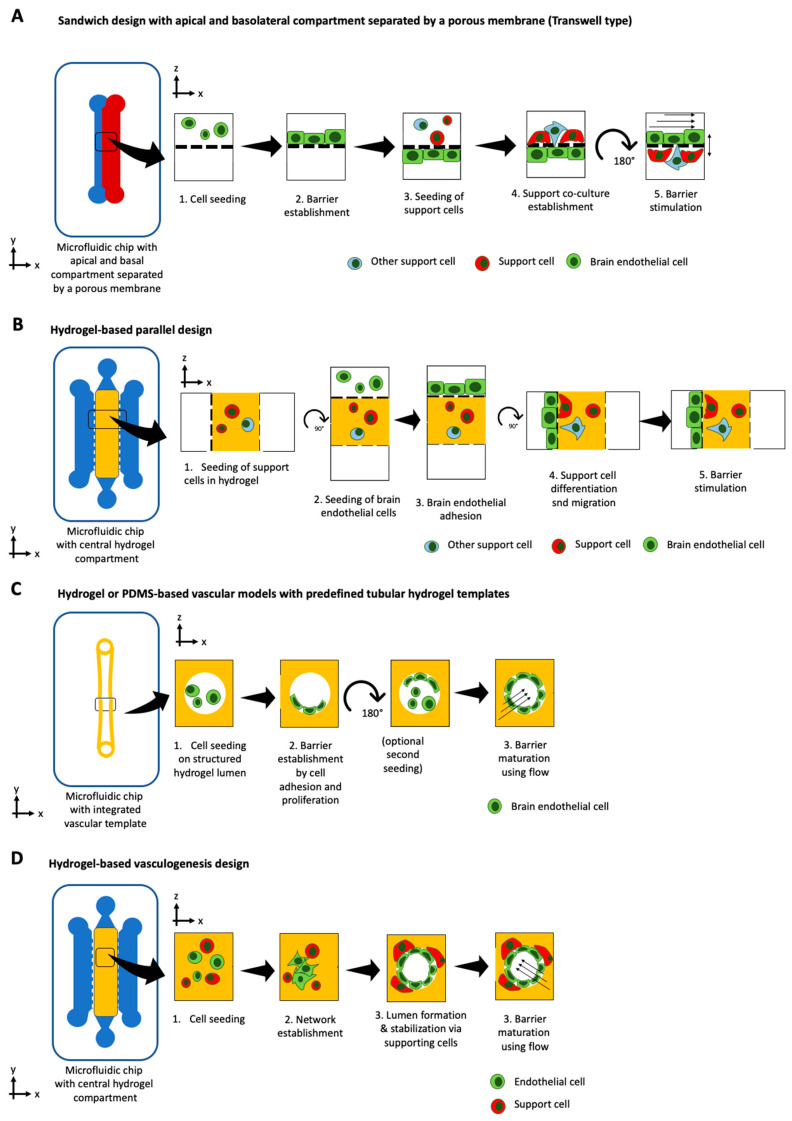
Basic principles for BBB-on-a-chip models using (**A**) tubular designs with a predefined tubular template, (**B**) hydrogel-based vasculogenesis design that promotes self-assembly of lumenized brain endothelial vessel structures with support cells, (**C**) sandwich Transwell design with co-cultures either on the bottom membrane side or bottom microchannel surface, or (**D**) parallel design similar to the vasculogenesis type, where the first support cells are embedded in the center hydrogel compartment and brain endothelial cells are consecutively seeded on one side of the hydrogel walls.

The blood-brain barrier (BBB) is a highly selective barrier, which ensures the separation of the circulating blood from the central nervous system (CNS). By strictly controlling the transport of molecules and ions, it is essential in maintaining cerebral homeostasis [167]. However, this complex selectivity also prevents potential therapeutics from reaching their cerebral target, rendering the BBB the biggest obstacle in cerebral drug delivery [168]. Therefore, many studies employing organ-on-chip technology have focused on unravelling the complexity of the BBB (see overview in Table 7). Over the years, four distinct chip designs within BBB-on-a-chip platforms have been developed, including the sandwich, the parallel, the tubular structure, and the vasculogenesis design (see Figure 4A–D). With an upper channel and a lower channel separated by a porous membrane, the sandwich design tries to recapitulate the traditional Transwell-type assembly. Usually, endothelial cells are seeded in the upper channel while astrocytes, pericytes, or other cells of the CNS are seeded in the bottom channel. Due to its configuration and the poor transparency of porous membranes, imaging capabilities are limited to advanced confocal imaging techniques. The emergence of transparent membrane materials such as PTFE have improved these limitations [169]. Falanga et al. employed transparent PET membranes for studying the effect of particle decoration with the membranotropic peptide gH625 and the iron-mimicking peptide CRTIGPSVC on the transport of nanoparticles across a murine BBB, based on b.End3 brain endothelial cells [170]. Brown et al. have adapted and improved the traditional sandwich design and generated a human neurovascular unit (NVU) model that allowed paracrine signaling of the co-cultured cells in a more physiological setup, by seeding brain microvascular endothelial cells on the bottom side of the membrane and introducing human pericytes, astrocytes, and neurons in a collagen hydrogel above [171]. In addition to an in vivo-like scaling, this study revealed a beneficial effect of pericyte conditioned medium on the integrity of the BBB at around 35 kOhms/cm^2^ and demonstrated the barrier protective properties of ascorbic acid, as well as the detrimental effects of glutamate. Recently, Maoz et al. have linked three sandwich chips to model the influx into the BBB, the brain parenchyma, and the efflux out of the BBB [172]. The linked NVU model was able to mimic metabolically critical physiological functions and cell phenotypes of the NVU, outperforming previous NVU in vitro models. Furthermore, the authors showed how methamphetamine exerted its effects on BBB influx rather than on the BBB efflux. Employing their human BBB-on-a-chip model based on iPSC-derived brain microvascular endothelial cells, Park et al. demonstrated the influence of hypoxia on the transport of chemicals and therapeutic antibodies (e.g., cetuximab) [173]. By exposing iPSCs to either normoxic or hypoxic conditions during differentiation into brain endothelial cells, it resulted in a more than five-fold increase in barrier tightness. In addition, selective transport of an anti-Transferrin receptor (TfR) antibody was demonstrated by perfusing antibodies at the vascular side of the hypoxia-differentiated BBB model. The more permeant MEM75 antibody was able to penetrate into the CNS channel, while retaining generally tight barrier properties. It is important to note that this clinically, highly relevant form of shuttling across the BBB could not be replicated in a standard Transwell set-up, due to its poor barrier function and high level of paracellular influx, underlining the importance of micro-physiological systems.

Within the parallel design, microchannels are aligned in parallel, replacing the need for a porous membrane. The parallel design, thereby, improves the two main disadvantages associated with the sandwich design: limited cell-cell interactions and poor imaging capabilities. By using a 3 µm gap micropillar array, Deosarker et al. were able to replace a porous track-etched membrane to separate neonatal rats’ endothelial cells and astrocytes [174]. This platform enabled direct cell-to-cell interactions and revealed the importance of astrocytes as well as astrocyte-conditioned medium on barrier integrity. By introducing breast cancer cells in the outer chamber, Terrell-Hall et al. [175] were able to show that cancer cells disrupt BBB integrity while the efflux transporter *p*-glycoprotein function remains intact. Similar to Brown et al. for the sandwich type, Adriani et al. expanded the design concept of the parallel design by connecting four channels in parallel, accounting for neurons, astrocytes, and endothelial cells and their respective media [176]. This 3D neurovascular chip supported cell-specific characteristics and demonstrated that the brain microvascular endothelial cell line hCMEC/D3 outperformed the umbilical vein endothelial cell line HUVEC for BBB applications. By using the MIMETAS OrganoPlate^®^, Wevers et al. [177] have enabled the study of antibody transcytosis in a human BBB model, that is capable of high throughput screening.

In contrast to the two rectangular design types mentioned above, the 3D tubular structure design provides cylindrical 3D microchannels and, thus, uniform shear stresses along the channel wall. By combining 3D printing with microneedles, Kim et al. were able to generate and cultivate mouse 3D brain microvessels based on b.End3 and mouse astrocytes for a period of three weeks, that recovered within four days after a D-mannitol-induced barrier disruption [178]. By coating a PDMS channel with collagen and seeding it with the immortalized rat brain endothelial cell line RBE4, Cho et al. developed a simple tubular BBB model for neuroinflammatory studies using TNF-α stimulation [179]. Unfortunately, ROS induction during oxygen-glucose-deprivation in an induced ischemia study did not show the expected regenerative capacity of Rho Kinase inhibition by Y-27632, and treatment with the antioxidant edaravone, which is a neuroprotectant used after acute ischemic stroke. Employing the fluidic technique of ‘viscous finger patterning,’ Herland et al. [180] generated a collagen hydrogel-based microchannel for the tubular co-culture of hBMVEC with human astrocytes or pericyte, respectively. Interestingly, throughout a three-day culture period, this tubular BBB model showed distinct differences in G-CSF and IL-6 secretion compared to Transwell cultures when challenged with TNF-α. Another fabrication technique for 3D tubular devices is two-photon lithography. Using this technique, Marino et al. have been able to produce tubular structures with geometries similar to those of capillaries. Due to its high printing resolution, bioprinting via two-photon polymerization, thus, provides a promising technique to recreate brain vasculature with in vivo-like geometries. By leveraging celullar self-assembly and self-organization, the vasculogenesis strategy constitutes an alternative approach in BBB organ-on-a-chip technology that focuses on the *de novo* formation of microvessels in vitro. In their study, Bang et al. not only generated a 3D self-organized BBB vascular network [181] with synaptic astrocyte interaction, but, in the process, determined optimal co-culture medium formulations. Recently, Campisi et al. [182] have published a 3D vasculogenesis model that encompasses human iPSC-derived endothelial cells, human pericytes, and human astrocytes in a microvascular network, capable of emulating physiological vessel geometries (lateral diameter lower than 50 µm). Overall, these four approaches can recapitulate basic functions of neurovascular and blood brain barrier functions with the biological and physiological advances lagging behind the vast progress in technological development and platform establishment. In addition, brain chips should not be expected to model and recapitulate the complex heterogenous architectures found in the human brain any time soon as this organ comprises a variety of highly specialized and localized anatomies including (please see comprehensive reviews, such as Matorakos and McGaven, for architectural details [183]), i.e., (i) meningeal and cortical vascular structures, (ii) capillary and postcapillary venules within brain parenchyma, (iii) fenestrated vessels in the choroid plexus as well as (iv) fenestrated vasculatures at the center of circumventricular organs (CVOs), and (v) traditional BBB architectures spanning around the perimeter of CVOs.

## 9. Central Nervous System-on-a-Chip: From Neurodevelopment to Drug Testing and Brain Disease

The central nervous system (CNS) constitutes the control panel of the human body. Characterized by its high complexity and plasticity in health and disease, the CNS has long been the subject of intense research. Organ-on-a-chip technology has provided a powerful new toolkit for the study of the CNS due to its ability to, at least in part, reconstruct specific structural features of the CNS’s highly complex architecture (see overview in Table 8). Among several areas of application, the recreation of neuronal networks using microfabricated platforms presents one of the largest. By introducing neurons and glial cells from multiple regions of the brain in a microfluidic chip and functionally connecting them, Dauth et al. have generated a novel, multiregional, neuronal network-on-chip, capable of demonstrating distinct properties of the hippocampus, the amygdala, and the prefrontal cortex [184]. The differences, which manifested themselves in aspects such as metabolism and electrophysiological activity changed upon functional connection, highlighting the importance of interconnectivity for studying physiological brain functions. Bang et al. were able to generate aligned neural circuits with visualizable synapses, thus, providing a microfluidic system with the potential applicability in CNS synapse research as well as neuro-muscular junction studies [185]. Similarly, Sances et al. recently employed microfluidics to study the effect of brain microvascular endothelial cells on the development of spinal motor neurons. Microfluidic vascular-neural co-cultivation led to enhanced neuronal function and in vivo-like signatures [186]. MacKerron et al. developed a microfluidic platform that can be used for the pharmacological profiling of CNS active compounds, based on an integrated calcium imaging readout [187]. Furthermore, the system might be of potential use in the study of neuromodulation and synaptic plasticity. Advances in both induced pluripotent stem cells (iPSCs) technology as well as organoid technology have enabled the generation of three-dimensional cellular constructs, capable of emulating both architectural and functional features of many organs, including that of the human brain in vitro [188]. While iPSC-derived organoids provide a powerful tool for modeling and understanding organ development, by recapitulating early developmental processes, differences still persist. These differences can mainly be ascribed to the lack or incorrect presentation of microenvironmental factors, such as biochemical and physical cues as well as multicellular interactions in a time-resolved and spatially-controlled manner. Combining organ-on-a-chip technology with iPSC technology, thus, provides a powerful strategic approach to address these limitations. By transferring embryoid bodies in a three-dimensional matrix within a microfluidic device, Wang et al. were capable of differentiating human cerebral organoids over a period of 33 days in perfusion [189]. These cerebral organoids not only displayed well-defined neural differentiation, regionalization, and cortical organization but exhibited an enhanced expression of cortical layer markers when compared to their static counterpart. In a follow-up study, Wang et al. could demonstrate that prenatal nicotine exposure causes a disruption in brain regionalization, as well as cortical development, coupled with abnormal neuronal differentiation and migration [190].

In their developmental study, Karzbrun et al. harnessed the advantages of microfluidics to study the physics and biology of early human brain development by revealing the mechanisms of wrinkling in a living system [191]. By introducing embryoid bodies into a micropillar array, Zhu et al. could demonstrate the advantages of microfluidics for the high content generation of human brain organoids for industrial applications [192]. While these studies could already demonstrate the beneficial effects of using organ-on-a-chip technology for brain organoid development studies, as of today, only a few studies have combined iPSC-derived brain organoids with microfluidics.

Due to the growing awareness that compounds, such as pesticides, can have detrimental effects on the health of the CNS, microfluidic platforms have been increasingly employed for toxicological applications. Recently, Koo et al. published a 3D tetra-culture platform for organophosphate toxicity screening [193]. This in vitro model was able to demonstrate the ability of organophosphates to penetrate through the BBB, subsequently inhibiting acetylcholinesterase activity and eliciting toxic effects, similar to those overserved in vivo. By interconnecting neurospheres and liver spheroids in a multi-organ-on-a-chip platform, Materne et al. have provided a potential tool for long-term substance testing that takes inter-organ cross-talk into account [194]. Exposure to the neurotoxic compound 2,5-hexanedione resulted in an increased sensitivity toward the compound for both neurospheres and liver spheroids upon co-culturing for 14 days, highlighting the importance of multi-organ approaches for toxicological studies.

A major focus of current organ-on-a-chip approaches is put toward the study of diseases of the CNS. To investigate nerve-cancer interactions in vitro, Lei et al. developed a high-throughput chip for potential drug screening applications [195]. The study revealed that neurons provide biophysical support to and guide directional movement of cancer cells. It was shown that cancer types with high perineural invasion display better migration capabilities along neurons and that, by specifically addressing pathways involved in neuron-cancer signaling, cancer migration could be inhibited in vitro. Fan et al. developed a 3D high-throughput screening platform for the generation of glioblastoma multiforme spheroids and subsequent administration of drugs via an integrated gradient generator [196]. In a follow-up study, the chip was used to demonstrate the beneficial effect of a combinatorial treatment of glioblastoma multiforme spheroids with the two clinical drugs, avastin and temozolomide, compared to temozolomide treatment alone.

The prevalence of neurodegenerative diseases, such as Parkinson’s disease and Alzheimer disease, and the concurrent lack of suitable treatment strategies have called for alternative disease models, capable of emulating disease onset and progression [197]. While the development of such CNS disease models progresses moderately, promising developments have been made. For instance, Park et al. developed a microfluidic array for the dynamic cultivation and subsequent exposure of 3D neurospheres to amyloid-beta, a peptide strongly associated with Alzheimer’s disease [198]. Dynamic cultivation resulted in larger spheres and robust neuronal networks, that were once exposed to the neurotoxic peptide displayed reduced viabilities, neural destruction, and synaptic dysfunction, which are all pathophysiological features of Alzheimer’s in vivo. Recently, Osaki et al. generated an amyotrophic lateral sclerosis model by combining three-dimensional skeletal muscle bundles with iPSC-derived motor neuron spheroids [199]. This system was not only able to demonstrate distinct differences between healthy and diseased motor neurons but, in addition, could prove that rapamycin and bosutinib cotreatment has considerable potential for ALS treatment.

## 10. Multi-Organ on-Chip Models: Combined Tissue Models to Screen Drug-Drug Interactions, Pharmacokinetics, and Pharmacodynamics

Drug-induced toxicities in liver, heart, kidney, and brain currently account for more than 70% of drug attrition and withdrawal from the market. Adverse drug reactions are often caused by off-target interactions or excessive binding of the drug to toxicity-prone cells [200]. Since inadequate pharmacokinetics (PK) and pharmacodynamics (PD) are key factors in drugs failure, interconnected multi-tissue cell culture models are considered to be a useful tool to investigate the pharmacokinetic profiles of drug molecules. For instance, drug-to-drug interactions were investigated by a combination of a mathematical PK–PD model and the experimental results using the multi-organ-on-a-chip (MOoC) as reported by Shinha [201]. In detail, the effects of metabolites of the anti-cancer pro-drug CPT-11 on lung cancer cells were determined. To evaluate DDI and resulting changes in drug concentration and metabolic capacity, the inhibitory capacity of simvastatin and ritonavir on the metabolism of CPT-11 was tested. The presented MOoC consisted of a pro-drug metabolizing liver part (HepG2 cells), a lung cancer part (A549 cells), and a stirrer-based micropump connected by micro-channels. Drug-specific parameters were determined by a combination of a simulated PK–PD model and the experimental results, showing comparable results and, ultimately, suggested that the combination of the PK–PD model and the MOoC is a useful way to predict adverse effects of drugs. In a more advanced, pneumatic, pressure-driven, four-organ system composed of intestine, liver, cancer, and connective tissue models, the effects of the anti-cancer drug 5-FU and the two pro-drugs of 5-FU (CAP and tegafur) were evaluated in terms of intestinal absorption, hepatic metabolism, and growth inhibition in cancer and connective tissue [202]. Additionally, Maschmeyer et al. integrated skin biopsies in a four-organ device consisting of liver, intestine, skin, and kidney model to recapitulate in vivo absorption, distribution, metabolism, and excretion behaviours [203].

Since cardiac and liver toxicity are the leading causes of post-approval drug withdrawals, these tissue types are of great interest to assess multi-organ drug interactions [204]. For instance, Oleaga et al. presented a multi-organ-on-a-chip platform, which allowed the analysis of a long-term cardiomyocyte and hepatocyte co-culture [205]. Using a MEA and a cantilever array, electrical activity and mechanical function of the cardiac cells could be determined and cardiotoxic effects of two drugs due to hepatic metabolism was observed. Similarly, a dual-organ-on-a-chip system was presented by Zhang et al. for automated analysis of an in vitro heart and liver organ model [206]. Biomarker (as albumin, glutathione S-transferase α, and creatine kinase MB) secretion was measured by functional electrodes, while environmental conditions (e.g., oxygen levels, temperature profiles) were observed. The drug testing capabilities and efficiency of the system were demonstrated by using acetaminophen and doxorubicin [207]. Moreover, Maoz et al. used a combination of TEER and MEA to enhance the analysis outcome of a myocardial barrier model [208]. Dynamic changes in this heart model, consisting of cardiomyocytes and a primary human endothelial cell monolayer, could be detected after challenging it with TNF-α as well as isoproterenol, which is a cardiac targeting pro-drug. The latest heart-on-a-chip developments involve emulating more complex, biological tissue-tissue interactions on a chip. Skardal et al. advanced the heart and liver organ-on-a-chip system by implementing a lung model in the same system [209]. The individual organ models were bio-printed and consisted of primary human hepatocytes, stellate cells, and Kupffer cells for liver organoids, induced pluripotent stem cells for heart organoids, and lung fibroblasts, epithelial cells, and endothelial cells for lung membranes. Analysis of the system was performed by measuring cardiac beating rates by real-time imaging, antibody-binding by impedance spectroscopy, and barrier function by TEER (as an addition to traditional supernatant analysis). When investigating potentially toxic effects of bleomycin on the lung organ model, adverse effects on the cardiac organoid was observed, which did not occur when treating the cardiac model alone with the same compound. This indicates the importance of multi-organ models to reveal interactions between multiple tissues and organs. Novak et al. demonstrated how an automated solution can investigate complex, vascularized, multi-organ systems with the group’s robotic interrogator and shows the potential of robotic solutions to solve the scalability of medium exchange as well as sampling of multi-organ systems [210]. As an application example, the system was used for long-term pharmacokinetic analysis of inulin-FITC for up to three weeks in an eight-organ system linked via vasculature and supported by computational PBPK modelling.

## 11. Conclusions

Organs-on-a-chip systems underwent a decade-long transformation and constant improvements to better emulate physiological or pathophysiological conditions, which already led to a deeper understanding of human physiology and disease progression at the micro-scale. While the majority of the organs-on-a-chip systems are used in academic settings, a small number of systems have shown real-world applications in the pharmaceutical industry, including CNS, vascular, heart, skin, liver, kidney, lung, and BBB models (see Table 1, Table 2, Table 3, Table 4, Table 5, Table 6, Table 7 and Table 8). The selected organ models have shown improved outcomes over existing Transwell cultures and used relevant bioactive molecules to demonstrate potential pharmaceutical applications. It is important to note that recent organ-on-a-chip progress has primarily been achieved by interdisciplinary approaches combining iPSC technology, cell self-assembly, microfabrication, and bioprinting to support cell-based microfluidics. Given the biological complexity of the described organ-on-a-chip system, future progress will likely be made in sensor integration to monitor key physiological parameters [18]. Overall, the current status of the described organs-on-a-chip system are summarized below.

*Lung-on-a-chip:* Among the first organs-on-a-chip to be developed, the basic chip designs did not change over the last decade besides some minor improvements in the stretching actuation and integrated sensing modules. Recent improvements relate to the integration of primary lung cell lines in combination with other cell types, such as endothelial cells, to mimic human pathologies and treatment. The apparent lack in technological progress can be associated with the excellent performance of the biochips capable of recapitulating important aspects and pathways of human lung pathologies, such as asthma, COPD, or lung cancer that are needed for drug screening.

*Skin-on-a-chip*: When looking into the progress of skin-on-a-chip systems, improvements are evident in the increased architectural features, such as inclusion of fat or nerve tissue as lipophilic drug reservoir, immune cells, and the nervous system. The application of skin-on-a-chip systems for pharmacological application lies mainly in the interconnection with other human tissues, where the skin compartment plays a role in transdermal transport, accumulation, and release of drugs and pro-drugs into a multi-organ-on-a-chip system comprising of hepatic metabolism, vasculature, and selected target tissues, such as kidney and heart.

*Liver-on-a-chip*: liver-on-a-chip systems have been shown to successfully mimic the enzymatic activity, spatial-organization, and anatomical along with ultra-structural features of the human liver. Consequently, future applications will involve the establishment of personalized disease models including relevant liver cell sources as primary hepatocytes and non-parenchymal cells to control structural, mechanical, and biochemical cues for investigating fundamental aspects of, e.g., non-alcoholic liver diseases (steatosis, cirrhosis, liver, and fibrosis) or alcoholic liver disease. For the production of next-generation multi-organ-chips, the establishment of liver-on-chip compartments is crucial since they play a leading role in the interconnection to other organs in terms of pharmacokinetics, drug metabolism, disease progression, and drug-induced hepatotoxicity.

*Kidney-on-a-chip:* chip-based kidney systems are still in its infancy due to technical and biological constraints on the recreation of the proper anatomical architecture of a single functional nephron comprising of tubules, glomeruli, arterioles, and venules. During the past years, however, progress is made to recapitulate the structural, mechanical, transport, absorptive, and physiological properties of the human kidney using an on-chip mono-culture (e.g., human podocytes) or co-culture models [211]. The bio-fabrication of functional kidney-on-chip systems for predicting drug efficacy and drug-induced kidney injury are, therefore, promising solutions for the detection of drug interactions and nephrotoxic effects in the near future.

*Heart-on-a-chip:* Similarly, heart models face the dilemma of being anatomically too inaccurate, considering the variety of functional structures of the human heart. However, their potential lies definitely in the creation of beating structures for drug screening and off-target cardiotoxicity studies, where the heart-on-a-chip system in combination with other tissues, such as liver and lung, shows promising progress.

*Vascularization-on-a-chip*: Vasculature-on-chip systems have demonstrated their applicability in multiple biological questions within the last decade. Existing techniques achieve meaningful vessel diameters and anatomies from arteries down to the capillary bed. However, the biggest challenge when using this vasculature as a conduit between organs in MOC still needs to be addressed. The integration of vasculature in place of microfluidic channels to connect organs would yield meaningful information on vessel health during drug exposure.

*BBB-on-a-chip*: While significant progress has been made in the development of highly complex BBB models, such as (i) the emulation of physiological shear forces, (ii) the integration of the cellular building blocks including astrocytes, endothelial cells, and pericytes, and (iii) the recapitulation of capillary morphologies in a self-assembled network, next generation BBB models will need to account for the drug target itself to further increase their applicability for pharmacological applications.

*CNS-on-a-chip*: Despite substantial advances in the modelling of the CNS employing microfluidic principles, efforts only recently ventured out to the recapitulation and interconnection of multiple regions of the brain as well as the integration of complex 3D tissues, such as brain organoids. With their ability to differentiate and reorganize into structural and functional analogues of the brain and its individual elements, next-generation CNS-on-a-chip models employing brain organoid technology will provide a powerful tool for neurodevelopmental, pathological, and pharmacological studies.

**Table 1:** Skin-on-a-chip applications.

Device Specifications				Assays	Additional Information		
Device Material	2D/3D	Application	Cell Type(s)	Assays	Special Feature	Outcome	Ref.
PDMS and PET porous membranes	Monolayer	Skin epidermis, inflamatory, edem	Human keratinocytes HaCaT, fibroblasts, Human umbilical vein endothelial cells (HUVECs)	Response to TNF-a (mRNA expression levels ELISA), Tight junction staining, leakage assay	Multi barrier competent Chip (3)	TNF-α-induced inflammation treatment with drug DEX	[74]
Polycarbonate cover-plates, PDMS-glass chip	Biopsy	Oral and ermal ubstance absorption	Prepuce samples	Stratified stratum corneum at the air–liquid interface, mRNA	4-organ-models for 28 days	Absorption, distribution, metabolism, and excretion	[203]
Hydrogel-based (collagen)	3D hydrogel Co-culture	Epidermis and dermis	Normal human dermal fibroblasts (NHDFs), normal human epidermal keratinocytes (NHEKs), Human umbilical vein endothelial cells (HUVECs)	Drug uptake on skin—concentration measured in the perfusion	Perfusion from within	Percutaneous absorption of caffeine and ISDN	[69]
PDMS and PC membrane	3D	Air-Skin-Interface	Fibroblasts and keratinocytes	Transdermal transport of FAM-tagged oligonucleotides	Air-Skin-Interfaces	Anti-proliferative role of doxorubicin	[73]
PDMS, membrane	3D	Differentiation	Fibroblasts and keratinocytes	H&E, Masson trichrome (MT), and Sirius red staining	Gravity flow	Skin differentiation on chip	[72]
PC, PMMA, PTFE filter membranes	3D	Skin permeation	N/TERT-1 keratinocyte, human primary foreskin-derived dermal fibroblasts	Diffusion of caffeine, testosterone, salicylic acid	Micro device vs. Franz diffusion cells	As good as Franz diffusion cells	[71]
PDMS, PMMA, PS, PET membrane	2D	Immune-competent	Human keratinocytes (HaCaT) human leukemic monocyte lymphoma cell line (U937)	TEER, lipopolysaccharides impact, UV, nickle sulfate, cobalt sulfate, glycerol, DNCB	Immune response, TEER on chip	ALI not possible	[75]
Commercial Biochip	2D/3D	Air-Skin-Interface	Murine fibroblasts (L929) and EpiDerm™ RhE.	TEER, impact of sodium dodecyl sulphate	Air-Skin-Interface, TEER on chip	Detection of tissue break down	[67]
PMMA	3D in fibrin dermal matrix	epidermal morphogenesis	Human primary foreskin-derived dermal fibroblasts	TEER, Raman, 2PP microscopy	orthokeratinized full-thickness SE within a microfluidic platform	Industrial close working chip design	[68]

**Table 2:** Lung-on-a-chip applications.

Device Specifics				Assays		Additional Information	
Device Material	2D/3D	Application	Cell Type(s)	Special Feature	Sensing Principle/Assay	Outcome	Ref.
PDMS	Monolayer, co-culture	Alveolar-capillary interface	human alveolar epithelial cells and microvascular endothelial cells	Stretchable membrane	TEER, albumin transport, neutrophil activation through e. coli, ROS, nanoparticle	First biomimetic device for breathing lung	[28]
PDMS	Monolayer	Pulmonary edema	Human pulmonary microvascular endothelial cells (Lonza), Alveolar epithelial cells NCI-H441	Stretchable membrane	interleukin-2 (IL-2) (for leakage of ALI = edema) vs. angiopoietin-1 and drug GSK219387	GSK219387 closes leakage from edema	[51]
PDMS, PE Membrane	Monolayer, co-culture	Disease COPD	Primary hAECs, human lung microvascular endothelial cells	ALI	interleukin-13 (IL-13) reconstituted the goblet cell hyperplasia, cytokine hypersecretion, and decreased ciliary function of asthmatics	Cytokines IL-8, M-CSF, IP-10, and RANTES healthy vs. COPD	[53]
PDMS, PET membrane	Monolayer, co-culture	Impact of smoking	Primary human small airway epithelial cell, microvascular endothelium	Cigarette smoke as ALI. Also e-cigarette.	upregulation of anti-oxidant heme oxygenase 1 (HMOX1) gene expression with smoke exposure phosphorylation of the antioxidant regulator Nrf2 ciliary beat frequency interleukin 8 (IL-8) secretion of COPD cells influenced by smoke	Smoking upregulates expression of oxidation-reduction	[45]
PDMS, PET Membrane, gold electrodes on PC	monolayer	TEER and barriers (also gut with CaCo^2^)	primary human airway epithelial cells (hAECs)	TEER of ALI	Application of chelating agent EGTA	EGTA leads to destruction of barrier	[60]
PDMS	3D organoids	Organ toxicity	Hepatic stellate cells (HSCs) (+), primary human hepatocytes (+), Kupffer cells (+), induced pluripotent stem cell-derived cardiomyocytes (iPSC CMs) (*), human primary cardiac fibroblasts (+), lung microvasculature endothelial cells (+), airway stromal mesenchymal cells (+), bronchial epithelial cells (+)	Cardiac beat rate measurement by real-time imaging and computational analysis, antibody-binding by impedance measurement, and barrier function by TEER measurement	acetaminophen, N-acetyl-L-cysteine on liver, epinephrine and propranolol on heart, Bleomycin	Interlinked responds of combined organoids	[209]
PDMS, PDMS membrane	Lung monolayer, whole blood HUVECS channel wall	Blood-air interface pulmonary thrombosis	HUVECs, human lung microvascular endothelial cells (HMVEC-L), blood	Blood for perfusion of vessel	Inflammatory cytokine-induced pulmonary thrombosis	LPS endotoxin indirectly stimulates intravascular thrombosis	[62]
PDMS, PDMS membrane	Monolayer	Blood-air interface	Bronchial epithelial 16HBE14o−cells, Primary human pulmonary alveolar epithelial cells (pHPAEC)	Mechanical strain of alveolar barrier during breathing	IL-8 concentration, viability	Stretching influencing the transport of small molecules	[56]
PDMS, flexible circuit board	Monolayer	Blood-air interface	Human type II alveolar epithelial-like A549 cells (–)	Mechanical strain of alveolar barrier during breathing	Barrier movement and membrane permeabilization sensing by real-time measurement of resistivity changes in three impedimetric coplanar electrodes	Monitoring in real-time the integrity of an epithelial barrier located at a distance of 1 mm	[57]
PMMA	Monolayer	Epithelial smooth muscle interface	Human airway epithelial cells (Calu-3), human bronchial smooth muscle cells (hBSMCs)	ALI	Device characterization, tight junctions	Stable hydrogel-based ALI	[65]
PDMS	Spheroids	photodynamic therapy (PDT)	A549 and non-malignant MRC-5	3D spheroids, 384 wellplate based	Verification of spheroid viability, ALA accumulation, ROS generation	High PDT effectiveness on lung spheroids required additional time after treatment.	[66]
PDMS, PET membrane, glass	Monolayer	Various barrier models, gut, and BBB	A549	TEER on chip	TEER, ZO1, b-cantenin, leakage assay	One device for different barrier models	[212]
PDMS, silicon	Monolayer	Lung inflammation	Beas-2B (ATCCsnumber: CRL-9609t) is a human bronchialepithelial cell line infected with an adenovirus (12-SV40 hybridvirus, Ad12SV40 peripheral blood mononuclearcells (PBMCs)	Fibrocyte migration	Cell migration, ELISA	CP, CXCL12, and CXCR4 responsible for fibrocyte extravasation in lung inflammation	[63]
PDMS, PLGA for membrane	Monolayer	Tumor invasion	A549, HFL1, and HUVECs	Electro spinning of PLGA membrane	Device characterization, f-actin, DAPI, Calcein-AM, CD31 expression of HUVECs	Simulate in vitro the tumor microenvironment alveolar biochemical factors	[64]
PDMS, glass, PET membrane	Monolayer	Lung	A549	TEER (Evom)	Surface tension influenced by surfactant secretion	Chip higher TEER	[58]
Paraffin, glass, PET, and PC membrane	Monolayer	Lung	Calu-3	TEER (Evom)	14C-sucrose permeability	Permeability higher through	[59]
PDMS, glass	Monolayer	Lung	A549	Phosphor lipids, lamellar bodies, F-actin	Formation of laminar bodies	Laminar body formation depends on shear	[61]

**Table 3:** Liver-on-a-chip applications.

Device Specifics					Assays		Additional Information	
Device Material	2D/3D	Culture Conditions	Cell Type(s)	Application	Staining	Assays	Outcome	Ref.
Polysterene (Nortis)	2D	Flow conditions, Collagen I	Primary rat and human hepatocytes	Hepatotoxicity	Calcein-AM, Ethidium-homodimer1, HNF4α	Lactate dehydrogenase (LDH) release, alanine aminotransferase (ALT), albumin CYP1A1/2, and CYP3A4 activities, visualization o canalicular structures	Exhibited higher viability and improved hepatic functions	[96]
PDMS	Bio-printed 3D spheroids	Spheroid culture encapsulated within photocrosslinkable Gelatin methacryloyl (GelMA) hydrogel	human HepG2/C3Aspheroids	Drug toxicity assessment	cytokeratin 18, MRP2 bile canalicular protein, and tight junction protein ZO-1	Albumin, alpha-1 antitrypsin, transferrin, and ceruloplasmin	Treatment with acetaminophen induced a toxic response in the hepatic construct that was similar to published studies on animals	[213]
PDMS	3D liver spheroids	Flow conditions, low-attachment spheroid microplates	HepaRG cells, 3D pancreatic islet microtissues	Pancreatic islet–liver crosstalk based on insulin and glucose regulation	Insulin, glucacon, cytokeratin 8/18, vimentin, albumin, CYP3A4	Albumin, glucose, insulin	Demonstrated a functional feedback loop between the liver and the insulin-secreting islet micro-tissues	[214]
3D Perfusion IbiTreat, (Ibidi)	2D and 3D	hyaluronan and poly(ethylene glycol) (HA-PEG), alginate and agarose hydrogels for 3D, PLL or HEP Coat coated for 2D	HepG2 and hiPS-HEP	Comparison of agarose, alginate, and HA-PEG hydrogels	-	Rheology, dextran diffusion	In HA-PEG hydrogels, 3D hiPS-HEPs showed an increased viability and higher albumin production compared to cultures in the other hydrogels	[95]
PDMS, PMMA, PC membrane	3D	Flow, conditions, Matrigel	HepG2, LX-2, EAhy926, U937	Investigation of pathophysiological process of individual non-parenchymal cells in alcohol-induced ALD	ZO-1, CD14 and F-actin, VE-Cadherin, and eNOS for EAhy926, VEGF, and α-SMA for LX-2	Albumin, urea, ROS	Alcohol damages the tight junction and reduces the release of NO of EAhy926 cells	[99]
PDMS	2D	static and flow, fibronectin and collagen I	Primary human hepatocytes, LX-2, EA.hy926, U937	Recapitulation of a liver sinusoid-on-a-chip	CD31	Albumin, urea, CYP3A4	Higher albumin synthesis (synthetic), urea excretion (detoxification) was observed under flow compared to static cultures.	[100]
PDMS	2D	static and flow conditions, co-Culture of primary rat hepatocytes and endothelial cells	Primary rat hepatocytes, primary rat adrenal medullary endothelial cells (RAMECs), bovine aortic endothelial cells (BAECs)	Analysis of viral replication for the hepatotropic hepatitis B virus (HBV)	-	Urea, analysis of secreted HBV, RT-PCR	A dual-channel configuration under flow condition seems to be the best long-term liver model and more closely mimics the structure and microenvironment of the liver sinusoid.	[102]
PDMS	2D/3D	Flow conditions, monolayer, collagen sandwich, 3D spheroids, bioreactor	Primary rat hepatocytes	Assessment of repeated dosing chronic hepatotoxicity	-	Oxygen concentration, shear stress, viability, urea, albumin, CYP1A2, CYP2B1/2, CYP3A2	Chronic drug response to repeated dosing of Diclofenac and acetaminophen evaluated in PIC were more sensitive than the static culture control.	[215]
PMMA	2D/3D	Flow conditions, dECM bioink	HepaRG, HUVEC	Vascular/biliary fluidic channels for creating vascular and biliary systems	MRP2, CK31, Cholyllysyl-fluorescein	Albumin, urea, CYP expression	Drug treatment in the chip was highly influential and that liver functions were disrupted during the culture	[216]
PDMS	3D	Liver microsome into 3-D hydrogel pillars	Rat liver microsomes	Analyzing the reaction kinetics inside the microfluidic chip by liver enzymes	-	P450 reactions, enzyme kinetic parameters (Km, Vm, Diffusivity)	Reaction rates were significantly lower than the solution phase mathematical model that showed a good correlation with the results	[93]
Glass (Micronit)	2D	Flow conditions, collagen I	Primary human hepatocytes, THP-1, LX-2, HMVEC, primary human PMNs, LSECs	ADME/TOX studies	F-actin, VE-cadherin, α-SMA	Bile efflux, permeability, ROS	Partial immunologic functions within the liver sinusoid, including the activation of LSECs, promoting the binding of polymorphonuclear leukocytes (PMNs) followed by transmigration into the hepatic chamber	[101]
PDMS	3D	Flow conditions, 3D spheroid culture	Primary rat hepatocytes, hepatic stellate cells	Investigation of hepatocyte-hepatic stellate cell interactions	Albumin, P450 reductase	Albumin, urea	Enzymatic activity of spheroids co-cultured for 8 days was greater than that of mono-cultured spheroids	[97]
PMMA, PDMS, PC membrane	3D	flow conditions, 3D spheroid culture	human HepG2/C3A	development of bio-artificial livers, disease modeling, and drug toxicity screening	ZO-1, MRP-2, CPR, NADPH-cytochrome P450 reductase	Albumin, urea, expression of CYP enzymes, phase II enzymes, hepatic nuclear receptors, hepatic transporters, bile canaliculi transporters	timesaving, efficient, and safe in situ perfusion culture of hepatic spheroids in 3D culture	[217]

**Table 4:** Kidney-on-a-chip applications.

Device Specifics		Assays		Additional Information		-		
Device Material	2D/3D	Culture Conditions	Cell Type	Application	Staining	Assay	Outcome	Ref.
PDMS	2D, human proximal tubule	Flow conditions, 2D on collagen type I	Human primary proximal tubular epithelial cells (PTECs)	Renal drug clearance and drug-induced nephrotoxicity	CD13, E-cadherin, aquaporin 1, prominin 2, uromodulin, KIM-1, Na+/K+ ATPase, γ-glutamyl transpeptidase (GGT)	γ-Glutamyl transpeptidase (GGT) activity, ATP assay, ammoniagenesis, glucose reabsorption 25-(OH)2, vitamin D3 metabolism	Morphological and functional phenotypes of proximal tubule epithelium in vivo	[110]
PDMS	2D human renal vascular–tubular system	Flow conditions, 2D on collagen I	/human fetal kidney tissue, endothelial cells (HUVECs and HKMECs), fetal kidney pericytes	Reabsorption of albumin and glucose	F-actin, VE-cadherin, E-cadherin, tubulin	Histology, dextran perfusion, blood perfusion, albumin -and inulin perfusion, glucose perfusion	Double-layer human renal vascular–tubular unit (hRVTU) enabled by a thin collagen membrane that replicates the kidney exchange interface	[112]
PDMS	Glomerular vascular system	Flow conditions, ECM with fibrinogen, 2 wt% gelatin, 2.5 mM CaCl_2_, and 0.2 wt% transglutaminase	hPSC-derived kidney pretubular aggregates	nephrotoxicity, tubular and glomerular disease, and kidney regeneration	DNA, MCAM, PECAM1, PODXL, PDGFRβ, TUBA4A, ATP1A1 (Na/K ATPase subunit-α1), collagen IV and LTL	Flow profile analysis, flow cytometry, qRT-PCR	Vascularized kidney organoids cultured under flow had more mature podocyte and tubular compartments with enhanced cellular polarity and adult gene expression ompared with that in static controls	[113]
Polysterene (Mimetas)	3D, proximal tubule microvillus	Flow conditions, ECM, Collagen I	Cortical kidney tissue, urine-derived kidney cells	Personalized medicine	ZO-1, Ezrin, CDH-1, tubulin,	Barrier integrity assayP-gp transporter assay, trans-epithelial transport assay	Tubuloid-derived cells can form leak-tight, polarized kidney tubules, enabling personalized transporter studies in tubuloids	[218]
Acryl chambers	2D, glomerular filter barrier	Flow conditions, collagen I on porous membrane 0.02 µm pore size	Immortalized podocytes	transmembrane pressure in glomerular filter membrane, with potential implications for drug development	Actin	Dextran filtration, qPCR	Dysfunction of renal filtration is correlated with the reduction of synaptopodin expression and disorganized actin cytoskeleton	[219]
Polysterene (Mimetas)	2D and 3D	Flow conditions, 3D ECM Collagen I	Immortalized proximal tubule epithelial cells (CiPTEC-OAT1)	Screenings assay for renal drug-transporter interactions	ZO-1, tubulin, pericentrin	Functionality of P-gp and MRP2/4	Increased accumulation of glutathione-methylfluorescein (GS-MF) was observed upon inhibition with a combination of PSC833, MK571, and KO143	[220]
PDMS	2D	Matrigel-coated, flow and static conditions	ureteric bud (UB) cells isolated from primary mouse embryonic kidneys cells	Fluid shear stress studies	Dolichosbiflorus agglutinin	qRT-PCR	Link between the fluid shear stress from the initiation of urine flow and the development and function of the kidney	[221]
PDMS	Kidney glomerular capillary wall	Flow conditions, Matrigel coated	PS cells (PGP1, IMR-90-1, and IISH3i-CB6) and ES cell (H9) lines	Podocyte differentiation	podocin	Whole-transcriptome analysis	Podocyte differentiation protocol for iPS cells, and the development of a glomerulus chip	[222]
COP	2D	Flow conditions PET membrane, coated with collagen type I	Renal epithelial cells (raTAL and NRK-52E)	Transepithelial reabsorption of NaCl	-	TEER	RaTAL cells might sense ion concentrations on either side and adjust tight junction permeability accordingly.	[223]

**Table 5:** Heart-on-a-chip applications.

Device Specifics					Assays	Additional Information	
Organ(s)	Device Material	Application	Cell Type	2D/3D	Sensing Principle	Outcome	Ref.
Heart and liver	PMMA, PDMS, titanium, platinum (electrodes)	Cardiotoxicity (primarily from hepatic cytochrome P450 (CYP) metabolism)	Human iPSC derived cardiomyocytes and human primary hepatocytes	2D monolayers	Multi-electrode array for electrical activity sensing and cantilever array for sensing of cardiac mechanical function	Culture period of 28 days with stable cellular function/viability. Cardiotoxicity of two known drugs due to hepatic metabolism could be predicted.	[224]
Heart	PDMS, titanium, palladium, gold (electrodes)	Cardiac biomarker secretion	Human embryonic stem cell-derived cardiomyocytes	3D cell-laden hydrogel structures	Creatine kinase (CK)-MB sensing by impedance measurements using an aptamer functionalized micro-electrode	Aptamers specific to cardiac damage markers could detect dose-dependent usage of drugs. Beating rate and cell viability confirmed these measurements.	[225]
Blood vessel, heart, liver	PDMS, polystyrene	Cancer metastasis	HUVECs, HepG2, and hPSC line BJ1D	3D cell-laden hydrogel structures	Cardiac beat frequency sensing by fluorescence microscopy and computational analysis of micro-cantilevers.	Simulation of cancer invasion and metastasis from tumor to downstream liver model.	[226]
Heart	PDMS, titanium, platinum (electrodes)	Barrier function and electrical activity of endothelialized myocardium	HUVECs and human iPSC derived cardiomyocytes	Barrier model	Barrier integrity and electrical activity sensing by TEER-multielectrode array measurements	Measurement of biological processes and drug effects by TEER and MEA, allowing analysis of barrier function, changes in cell layer conformation, and electrical activity.	[208]
Heart and liver	PDMS, gold, silver (electrodes)	Organ toxicity	Human iPSC derived cardiomyocytes and HepG2/C3A hepatocellular carcinoma cells	3D organoids	pH sensing by light absorption of phenol red, oxygen sensing by fluorescence measurements of quenching effects of oxygen sensitive ruthenium dye and immunosensing by functionable electrodes	Platform for automated measurement of pH, oxygen, temperature, and biomarkers and microscope imaging over a period of 5 days of a dual organ-on-a-chip system with and without drug inducement.	[207]
Heart	PDMS, carbon (electrodes)	Beating behavior, cardiotoxicity	Cardiomyocytes isolated from neonatal Sprague Dawley rat pups	3D cell-laden hydrogel structures	Generated force sensing by fluorescence measurement of deflection of cantilevers	Generated cardiac microtissues showed that mechanical response (dynamic response, spontaneous stress) to compounds (isoproterenol and digoxin) can be used for drug testing	[227]
Heart	PDMS	Beating behavior, cardiotoxicity	Human iPSC derived cardiomyocytes	Small (100 μm height) 3D cell-laden hydrogel structures	Beat rate, conduction velocity, and field potential duration were measured by field potential analysis using commercially available MEAs	The micro-molded gelatin system with incorporated MEAs is able to measure electrophysiological changes in the cardiac tissue. Beat rate responded accordingly to drug (isoproterenol) application.	[228]
Heart, liver, and lung	PDMS, PMMA, gold (electrodes)	Organ toxicity	Hepatic stellate cells, primary human hepatocytes, Kupffer cells, iPSC-derived cardiomyocytes, human primary cardiac fibroblasts, lung microvasculature endothelial cells, airway stromal mesenchymal cells, and bronchial epithelial cells	3D organoids	Cardiac beat rate measurement by real-time imaging and computational analysis, antibody-binding by impedance measurement and barrier function by TEER measurement	The impact of multi-organ crosstalk is displayed by the effects of bleomycin on the cardiac tissue with and without a lung tissue present.	[209]
Heart, liver, skeletal muscle, and neuronal network	PDMS, PMMA, Titanium, platinum (electrodes)	Organ toxicity	Human hepatocellular carcinoma HepG2/C3A, human iPSC derived cardiomyocytes, human skeletal myofibers, human motorneurons differentiated from human spinal cord stem cell line (hSCSC) and human iPSC dervied cortical-like neurons	2D monolayers	Cardiomyocyte contraction (force) sensing by cantilever deflection (laser beam reflection) ^10, 11^ and electrical activity of cardiomyocytes or motoneurons by a multielectrode array ^11^	The system could detect influences of organ-cross talk in drug testing over a period of 14 days. ^10^ The 4-organ-system revealed stable conditions for all organs over a period of 28 days, making it a good fit for drug testing to investigate repeat dose toxicity. ^11^	[229]
Heart	Petri dish as housing for MEA, titanium (electrodes)	Beating behavior	Human embryoid stem cell line CCTL14 and human iPSCs	3D organoid	Cardiomyocyte beating force sensing by multi-electrode array and atomic force microscopy measurements	The system was able to determine the relation between cardiac beating behavior and correlated force in case of Duchenne Muscular Dystrophy.	[208]
Embryoid body (cardiac cells)	Glass, PEDOT:PSS (electrodes)	Autonomous beat rate of embryoid bodies	Mouse embryonic stem cells (mESC) derived cardiomyocytes	3D embryoid body	Cardiac beat rate sensing by voltage and displacement current measurement by large area electrodes	The system could measure the beating rate of whole embryoid bodies due to a combination of large, high capacity electrodes and usage of the displacement current measurement technique.	[230]
Heart	Glass, PDMS, poly(N-isopropylacrylamide)(PIPAAm), platinum (electrodes)	Muscle contraction	Cardiac ventricular myocytes harvested from two-day old neonatal Sprague-Dawley rats	2D monolayer	Cardiac tissue stress/contraction sensing by measurement of deformation of cells on elastic, thin film	Measurement of spontaneous contraction of cardiac tissues due to the impact of epinephrine.	[27]
Heart	PDMS, copper (force probe)	Muscle contraction/strength	Myocytes isolated from 0–2-day-old neonatal Sprague-Dawley rats, cardiac fibroblasts, and a transfected human embryonic kidney (HEK) 293T	3D cell-laden hydrogel fibers	Force sensing of the muscle strips by usage of a copper force probe, which applied force/deformation prior to the illumination. The deformation prior to and after the illumination was compared	The system was able to individually activate and pace the muscle fibers. With the addition of multiple fibers, the force can be graded. This offers a non-invasive way (compared to electrodes) for muscle stimulation.	[25]
Heart	Glass, PDMS, poly(N-isopropylacrylamide)(PIPAAm), aluminum, platinum (electrodes)	Muscle contraction	Cardiac myocytes extracted from ventricles of 2-day-old neonatal Sprague-Dawley rats	2D monolayer	Stress sensing by visual observation of cantilever deformation due to cardiac contraction.	Diastolic and systolic stress of cardiac tissues could be assessed and electrically stimulated with and without drug exposure.	[129]
Heart	PDMS, polystyrene beads (cyclic strain measurement),	Cardiac differentiation under cyclic strain	Neonatal rat cardiac cells and human iPSC-derived cardiomyocytes	3D cell-laden hydrogel structures	Cellular property sensing by immunofluorescence (live/dead, histochemistry) and scanning/transmission electron microscopy	The device showed that by applying cyclic strain, the cell-cell and cell-matrix interactions are better, and also micro-tissues displayed early synchronous beating.	[126]
Heart	Dextran, elastollan, silver flakes, PDMS	Long-term non-invasive measurement of contractile strength of cardiac tissues	Neonatal rat ventricular myocytes (NRVMs) and human iPSC-derived cardiomyocytes	Multiple 2D monolayers	Contractile stress and beat rate sensing by relative resistance changes of embedded sensors	3D multi-material printing allowed the creation of a device for non-invasive measurement of contractile stress development over a period of 4 weeks.	[130]
Heart	PDMS, glass, poly(3,4-ethylenedioxythiophene) carbon nanotube (PEDOT-CNT) (electrodes); UV crosslinkable methacrylated gelatin (GelMA)	On-chip culture of human 3D myocardium using cells from a single patient	Human iPSC-derived myocardial cells and endothelial cells	3D cell-laden hydrogel structures	Cell population alignment sensing by microscopy and beating frequency sensing by measurement of extracellular membrane potential	Usage of two cell types derived from the same hiPSCs this device mimics the myocardium of an individual human. Integration of hydrogel allowed 3D structuring and analysis was performed by integrated micro-electrode arrays and standard cell culture analysis.	[121]
Heart	PDMS, matrigel, fibrinogen, thrombin	Contractile activity	Neonatal rat heart cells	3D cell-laden hydrogel structures	Contractile force and beat rate measurement by video-optical measurement of pillar deflection	The device was able to measure the impact of drug and toxic substances on the beat rate of heart constructs, allowing a fast and simple screening method.	[127]
Heart	Polydopamine (PDA), polycaprolactone (PCL) (nanofibers), gelatin (cantilever), TiO2, and Ag (nanoparticels)	Contractile activity	Neonatal rat ventricular myocytes (NRVMs) isolated from 2-day-old Sprague Dawley rats	2D monolayer	Beat rate sensing by optical detection of cantilever deflection	By using 3D fiber scaffolds, the device supported the formation of anisotropic and contractile cardiac tissues. Non-invasive measurement of nanoparticle impact enables good drug screening capabilities.	[128]
Heart	Quartz (substrate), gold (electrodes)	Beating behavior	Neonatal rat cardiomyocytes	2D monolayer	Beat rate and amplitude sensing by impedance detection	The device can measure the mechanical contraction status of the cardiac tissue, which can be linked to the pumping of blood and plays a vital role in drug screening for heart medication.	[123]
Heart	PMMA, alginate, gelatin methacryloyl (GelMA), photoinitiator Irgacure 2959 (bioink)	Contractile activity	HUVECs, GFP-labeled HUVECs, and neonatal cardiomyocytes isolated from two-day-old Sprague-Dawley rats	3D bioprinted endothelial structure with 2D cardiac monolayer on top	Beat rate and amplitude sensing by optical microscopy and computational analysis	The endothelial cells in the bio-printed fibers of the scaffold would migrate toward the edge of the fibers and form a layer of confluent endothelium, mimicking a blood vessel. In combination with the myocardium and a perfusion bioreactor, this was then used for drug screening.	[132]
Heart	PEG-silane, PDMS, silicon (cantilever)	Electrical and contractile activity	Human cardiomyocytes derived from the hESC-10-0061 stem cell line	2D monolayer	Electrical activity sensing by using an MEA and contractile stress sensing using cantilevers deflection measured by a photodiode laser and a photodetector.	The system is able to detect electrical and contractile activity of cardiac tissues and both sensing areas can be upscaled.	[131]
Heart	PDMS, glass	Cell proliferation/viability increase in 3D vs 2D	Human primary cardiomyocytes	3D cell-laden hydrogel structures, 2D monolayer	Cell proliferation and viability sensing by fluorescence microscopy	The device showed that the proliferation rate was higher in 3D and the negative effects of isoproterenol was lower in 3D.	[122]

**Table 6:** Vascularization-on-chip applications.

Device Specifics						Assays	Additional Information		
Method	Device Material	Hydrogel	Concentration	Cell Source	Cell Type(s)	Assays	Application	Outcome	Ref
Microchannel patterning	PDMS	Collagen 1	0.1 mg/mL (coating)	Primary	HUVEC	Live/dead staining Immunofluorescence staining Blood perfusion	Thrombosis	Thrombosis-on-chip model created by PDMS molding of 3D-printed healthy and stenotic vessel	[139]
	PDMS	PDL/fibronectin	1 mg/mL (coating)	Primary	VSMC HUVEC	Immunofluorescence staining	Platform for blood vessel-related diseases	Successful cultivation of SMC and HUVEC on micro-wrinkled PDMS	[140]
	PDMS	Fibronectin	250 µg/mL	Primary	HUVEC	Immunofluorescence staining	Identify combinatorial effect of fluid flow and substrate rigidity on endothelial cells	Pronounced cell response to flow-induced shear stress and underlying substrate mechanics	[141]
	Hydrogels	Alginate Gelatin Gel-MA	1–5% 1–9% 1–9%	Primary	HUVEC	Diffusional permeability	Enhancing physiological relevance via microfabrication of hydrogels	Hydrogel-based microfluidic device as a vessel-on-chip model	[143]
	Gelatin	Gelatin	9%	Primary	HUVEC	Immunofluorescence staining Diffusional permeability	Circular microfluidic channels	Fabrication of branched vascular networks in a hydrogel-based microfluidic device	[144]
	PDMS	Collagen 1	2.5 mg/mL	Primary	HUVEC SMC Neutrophil	Diffusional permeability Leucocyte transmigration Blood perfusion	Atherosclerosis	Formation of pathophysiological architectures for real-time study of cardiovascular disease	[146]
	PDMS Polycarbonate	Fibrin	Scaffold	Primary	HUVEC lrVEC hMSC	Endothelial sprouting Blood perfusion Leukocyte adhesion and migration	Vascularization of tissue engineered scaffold	Engineering of vasculature in novel polycarbonate scaffold with subsequent surgical anastomosis	[147]
Sacrificial molding	PDMS	Collagen 1	3 mg/mL	Primary	HUVEC hBMSC/hLF/ hASMCs/ hKPC	Diffusional permeability Immunofluorescence staining Western blot RT-qPCR	Barrier function variation with inflammatory exposure	Identification of key proteins in regulating barrier function in inflammation	[153]
	PDMS	Collagen 1	2.5 mg/mL	Primary	hMVEC	Diffusional permeability Immunofluorescence staining RT-qPCR	Influence of hemodynamic shear stress on endothelial barrier function	Identification of novel mechanosensory complex	[231]
	PDMS	Gel-MA	4–16%	Primary cell line	HUVEC rSMC 3T3 fibroblast	Live/Dead staining Immunofluorescence staining Diffusional permeability Mechanical compression	Vascularization of tissue engineered structures	Engineering of all three layers of a mature blood vessel	[232]
	PDMS	Collagen 1	3 mg/mL	Primary	HUVEC hASMC	Metabolic activity Immunofluorescence staining	Defining physiological vessel functionality or pathologic remodeling	Formation of stable arteriole and artery-like structures	[151]
	PDMS	Collagen 1	6.5 mg/mL	Primary	hMVEC	LifeACT actin remodeling Diffusional permeability Immunofluorescence staining Live/Dead staining	Model for low-intensity anti-vascular ultrasound therapy	Efficacy of low-intensity ultrasound treatment of tumors dependent on fluid flow	[149]
	PDMS	Collagen 1	3 mg/mL–6 mg/mL	Primary	hLEC/HUVEC nhMF/CAF	Live/Dead staining Cytokine quantification RT-qPCR Diffusional permeability Immunofluorescence staining	Lymphatic vessel model for understanding physiologic and pathology	Organotypic lymphatic function dependent on cell-to-cell signaling	[154]
Self-assembly	PDMS	Collagen 1	3 mg/mL	Primary	HUVEC	Sprouting analysis Immunofluorescence staining Calpain and MMP inhibition	Platform to investigate hemodynamic and biochemical factors in angiogenesis	Angiogenesis dependent on IF and VEGF concentration	[159]
	PDMS	Fibrin	2.5 mg/mL fibrinogen 1 U/mL Thrombin	Primary	HUVEC hLF	Sprouting analysis Immunofluorescence staining	Flow-mediated endothelial dynamics and phenotype changes	Interstitial flow regulates angiogenic sprouting and endothelial cell phenotype	[158]
	PDMS	Fibrin	2.5 mg/mL fibrinogen 1 U/mL thrombin	Primary	HUVEC hASC	Network quantification	Direct and indirect perfusion of cell-seeded hydrogels	Direct cell-to-cell contact as well as reciprocal signaling molecules play a vital role in vasculogenesis	[8]
	PDMS	Fibrin	2.5 mg/mL fibrinogen 1 U/mL thrombin	Primary	hMVEC-dLyAd NHLF	Sprouting analysis Immunofluorescence staining Cell viability	Lymphangiogenesis in tumor formation	Interstitial flow augments lymphatic endothelial sprouting in synergy with lymphangiogenic factors	[160]
	PDMS	Fibrin	2.5 mg/mL fibrinogen 1 U/mL thrombin	Primary	HUVEC NHLF	Diffusional permeability Glycocalyx staining Colocalization analysis Transcytosis mechanism	Drug transport across the vascular endothelium and into the target tissue	On-chip model recapitulates physiologic paracellular and transcellular permeability	[161]
	PDMS	Fibrin	5 mg/mL fibrinogen 1 U/mL thrombin	Primary	HUVEC NHLF	Immunofluorescence staining Cell viability Network quantification Diffusional permeability Colocalization analysis	Vascular remodeling in Idiopathic pulmonary fibrosis	Anti-vasculogenic drug acts on endothelial network formation and endothelial–perivascular interactions	[162]
	PDMS	Fibrin	2.5 mg/mL fibrinogen 0.5 U/mL thrombin	Primary	HUVEC NHLF hPC-PL	Blood vessel contraction Oxidative stress Immunofluorescence staining Diffusional permeability	Nanoparticle toxicity on endothelial barrier	Nanoparticle exposure leads to microvessel contraction and nanoparticles are transported via caveolae-mediated transcytosis	[163]
	PDMS	Fibrin	1 U/mL thrombin	Primary	HUVEC NHLF	Immunofluorescence staining Diffusional permeability	Pathogenesis of inhaled atmospheric nanoparticles	Atmospheric nanoparticle inhalation leads to a loss of tight junctions and increased vascular permeability	[164]

**Table 7:** Blood-brain-barrier-on-a-chip applications.

Device Specifics					Assays		Additional Information				
Method	Device Material	Culture Condition	Cell Type	Immuno-Staining	Fluorescent Tracker [kDa]	TEER Values	Other Features	Shear Stress [Pa]	External Stimuli	Outcome	Ref.
Sandwich Design	PDMS 3D	17 days ECM: laminin	Primary hBMVEC (human) iPSC astrocytes pericytes	ZO-1 + Phalloidin staining	Dextran: 10, 70	35,000 Ω	Ascorbate transport	2 × 10^−3^	Cold shock Glutamate	Pericyte conditioned medium enhances BBB integrity	[171]
Sandwich Design	PDMS 2D	7 days ECM: Col IV, fibronectin, Col I	b.End3 (mouse) C8D1A (mouse)	Claudin-5	Dextran: 70	-	-	0.5	-	Optically transparent PTFE membrane might be a suitable alternative	[169]
Sandwich Design	PDMS 2D	6 days ECM: Col I/IV	hCMEC/D3 or primary rat endothelial cells primary pericytes (rat) primary astrocytes (rat)	ZO-1 β-catenin	Dextran: 4.4 Albumin: 67 Sodium fluorescin	175 Ω cm^2^	Direct contact between endothelial cells and pericytes	1.5 × 10^−2^	-	Primary cells exhibited better BBB properties than hCMEC/D3 cells.	[212]
Sandwich Design	PMMA 2D	7 days ECM: -	bEnd.3 (mouse)	Claudin-5	-	1150 Ω cm^2^	Internalization of nanoparticles	1.5 × 10^−2^	-	GH625 peptide modification on nanoparticle facilitates the transport across the BBB	[170]
Sandwich Design	Objet VeroClear photo-polymer - Parylene-C coating 2D	10 days ECM: Col I Fibronectin	iPSC derived BMEC (human) primary astrocytes (rat)	ZO-1 Claudin-5	Dextran: 4, 20, 70	2000 Ω cm^2^	Caffeine Cimetidine Doxorubicin	1.4–25 × 10^−3^	-	First time the application of iPSCs-derived BMEC in co-culture with astrocyte (enhanced BBB integrity) Doxorubicin disrupts BBB integrity after 24-h treatment	[233]
Sandwich Design	PDMS and polycarbonate 2D	4 days ECM: Fibronectin vs. Matrigel	Primary BMVEC (mouse) Astrocytes (mouse)	ZO-1 GFAP	Dextran: 3, 7, 10	3500 Ω	-	0.1–3	Histamine disruption	Multi-channel model with an integrated electrical impedance sensor array Matrigel provides better barrier integrity than fibronectin	[234]
Sandwich Design	PDMS 2D	6 days ECM: Fibronectin and Col IV	bEnd.3 (mouse)	Claudin-5	Dextran: 4, 20, 500	172 Ω cm^2^	-	0.1–0.6	-	Angiopep-2 peptide modification facilitates nanoparticle transport across the BBB	[235]
Parallel design	PDMS 3D	5 days ECM: fibronectin	RBE4 (rat) astrocytes (rat)	ZO-1 GFAP	Dextran: 40	250 Ω cm^2^	-	3.8 × 10^−4^	-	Co-culture with astrocytes increases barrier integrity Cell/cell interactions are observed Endothelial cells are sensitive to astrocyte conditioned medium	[174]
Parallel design	PDMS 3D	7 days ECM: Col I	HUVEC/hCMEC/D3 (human) primary astrocytes (rat) primary neurons (rat)	ZO-1 VE-cadherin GFAP DCX	Dextran: 10, 70	-	Glutamate transport Calcium imaging	-	-	hCMEC/D3 shows significantly higher barrier integrity compared with HUVEC Neurons in triple co-cultures grow progressively in 3D Col I hydrogels	[176]
Parallel design	PDMS 3D	4 days ECM: Fibronectin	HUVEC (human) astrocytes (rat) Met-1	-	Dextran: 3, 70	-	Efflux activity (Rhodamine 123)	1.9 × 10^−4^		Brain tumor model disrupts BBB integrity but retains P-gp activity	[175]
Parallel design	OrganoPlate Mimetas Polystyrene 3D	5–9 days ECM: Col I	TY 10 (human) hBPCT (human) hAst (human) Immortalized cell lines	VE-cadherin Claudin-5 PECAM-1	Dextran: 20	-	Antibody transcytosis	~0.12	-	Applicable to HTS	[177]
Tubular design	PDMS 3D	3 days ECM: Col I	RBE4 (rat) Human neutrophils	ZO-1 VE-cadherin	Dextran: 40	-	Transmigration of neutrophils	-	TNF-α/OGD	TNF- α elevates the release of several cytokines OGD induced the ischemia model ROCK inhibition restores BBB integrity	[179]
Tubular design	PDMS 3D	3 days ECM: Col I/IV	hBMVEC (human) astrocytes (human) or pericytes (human)	ZO-1 VE-cadherin GFAP SMA	Dextran: 3	40–50 Ω cm^2^	-	-	TNF-α	Co-culture with pericyte or astrocyte enhances BBB integrity Distinct differences in G-CSF and IL-6 secretion level between Transwell and chip cultures	[180]
Tubular Design	PDMS 3D	3 days ECM: Col I	Primary BMEC (rat) primary astrocyte (rat)	ZO-1 VE-cadherin	Sodium fluorescein	1298 Ω cm^2^	1.Expression level of P-gp and Glut-1 2.Modeling of extravasation in brain metastasis 3. Drug screening	1 × 10^−2^	-	Co-culture enhances BBB integrity U87 were unable to cross the BBB Only temozolomide is able to pass through BBB and kill U87 Applicable to HTS	[236]
Tubular Design	PDMS 3D	4 days ECM: Col I Matrigel HA	hCMEC/D3 (human) astrocytes (human)	ZO-1 GFAP	Dextran: 4	1000–1200 Ω cm^2^	TEM	0.07	TNF-α	Shear stress significantly improves BBB integrity Pulsatile flow drives retrograde transport along the basement membrane	[237]
Tubular Design	IP-DiLL -SU8 3D	3 days ECM: -	b.End.3 (mouse) U-87 (human)	ZO-1	Dextran: 10	71 Ω cm^2^	-	-	-	Two-photon lithography fabricated 3D tubular structures with 1:1 scale of capillaries	[238]
Vasculogenesis Design	PDMS 3D	3 days ECM: Fibrinogen	iPSC-EC (human) brain pericytes (human) brain astrocytes (human)	CD31 ZO-1 GFAP	Dextran: 20, 70	-	-	-	-	The best combination of cells for co-culture is identified	[182]

**Table 8:** Central nervous system-on-a-chip applications.

Device Specifics				Assays	Additional Information			
Application	2D/3D	Device Material	Cell Type	Assays	Flow	External Stimuli	Outcome	Ref.
Neuronal development-3D Organoid formation on chip	3D ECM: Matrigel	PDMS	iPSCs	Immunocytochemistry PCR TUNEL assay	25 µL h^−1^	-	Organoids display feature specific identities such as neuronal differentiation, brain regionalization, and cortical spatial organization Improved cortical development compared to static culture conditions	[189]
Neuronal development-Prenatal nicotine exposure	3D ECM: Matrigel	PDMS	iPSCs	Immunocytochemistry PCR TUNEL assay Neurite outgrowth assay (TUJ1)	25 µL h^−1^	Nicotine	Nicotine exposure led to premature neuronal differentiation, disrupted brain regional organization, abnormal cortical development, neuronal outgrowth, and increased cell apoptosis.	[190]
Neuronal development-Effect of endothelial cells on neuronal development	2D	PDMS	iPSC derived spinal motor neurons iPSC derived BMECs	Immunocytochemistry Transcriptomics Live Calcium Transient Imaging Analysis	-	-	Vascular-neural interaction and specific gene activation enhances neuronal function and enables in vivo-like signatures	[186]
Neuronal development-High throughput generation of human brain organoids	3D ECM: Matrigel	PDMS	-	Immunocytochemistry PCR	-	-	Controlled formation of human brain organoids on chip	[192]
Neuronal development-Neuronal differentiation and chemotaxis	3D	PDMS	hNT-2 hNPCs Immortalized hBMECs	Immunocytochemistry Chemotaxis assay	-	CXCL12 SLIT2-N	hNPCs respond to shallow gradients of CXCL12 only in the presence of a micro-environment mimicking the brain parenchyma milieu hNPCs are more polarized in the presence of a neuronal-glial cell population, which leads to more directed movement	[239]
Neuronal development-Physics of brain folding	3D ECM: Matrigel	PDMS	hESC	Immunocytochemistry RNA sequencing AFM	-	Blebbistatin	On-chip organoid approach successfully mimics the early developing cortex Lissencephalic organoids display reduced convolutions, modified scaling, and a reduced elastic modulus	[191]
Neuronal networks - Multi-regional brain on chip	2D	PDMS	Hippocampal neurons (rat) Amygdala neurons (rat) Prefrontal cortex neurons (rat)	Immunocytochemistry Proteomics MS Oxygen Measurement MEA		Phencyclidine hydro chloride	Distinct differences in metabolism in different brain regions in vitro Changed electrical activity profile upon connecting individual regions PCP treatment alters electrical activity in a region-dependent manner	[184]
Neuronal networks - 3D printed nervous system	3D	Silicone Polycap-rolactone	Hippocampal neurons (rat) SCG neurons (rat) Schwann cells (rat) PK-15 cells	Immunocytochemistry Viral assay	-	Pseudorabies virus	Interconnectivity in-between individual cellular components Schwann cells and hippocampal neurons are refractory to axon-to-cell infection of pseudorabies virus	[240]
Neuronal networks - 3D neural circuit	3D ECM: Matrigel	PDMS	Cortical neurons (rat)	Immunocytochemistry Calcium imaging	-	-	Axons formed in vivo like neural bundles with fasciculation and defasciculation. Aligned neural circuits with visualizable synapses.	[185]
Neuronal networks - Neuronal network for active compound testing	2D	PDMS	Hippocampal neurons (rat)	Immunocytochemistry Viability assay Calcium imaging	0.4–10 µL min^−1^	Glutamate	Integration of microfluidic perfusion with Ca^2+^ imaging techniques	[187]
Neuro-degenerative diseases - Alzheimer’s disease	3D neurospheroid	PDMS	Primary cortical neurons (rat)	Immunocytochemistry CCK-8 SEM	Interstitial fluid flow	Amyloid β	Interstitial fluid flow positively affects the maturation of neurospheroids Amyloid β treatment induced neural network induction	[198]
Neuro-degenerative diseases-ALS	3D ECM: Col I	PDMS	iPSCs derived motor neurons iPSCs derived skeletal muscle cells iPSCs derived endothelial cells	Immunocytochemistry PCR Ca^2+^ oscillation imaging SNP genotyping Whole exome sequencing Western blot	-	Rapamycin Bosutinib	Muscle contraction could be induced by MN activity once NMJ is formed ALS motor unit displayed higher levels of apoptosis and reduced muscle contraction force Combinatorial treatments improved neuronal survival and an increased muscle contraction force	[199]
Brain cancer chip for HTS	3D	PEGDa	U87 Primary glioblastoma tumors	Immunocytochemistry Diffusion test Viability	-	Pitavastatin Irinotecan Temozo-lomide Bevacizumab	HTS platform for spheroid formation and drug screening Combinatorial treatment with TMZ and BEV were more effective on glioblastoma spheroids	[195,241]
Cancer migration study	-	PDMS	Hippocampal neurons (rat) Cortical neurons (rat) DRG neurons (rat) PC-3 (human) Panc-1 (human) MCF-1 (human)	Immunocytochemistry	-	β -blockers muscarinic antagonists Neuron injury (6-hydroxy-dopamine)	Neurites guide the directional movement of cancer cells. Cancer cells with high levels of perineural invasion display greater migration along neurites. Neuron injury reduces migration of cancer cells. Muscarinic antagonists reduced migration.	[236]
Effects of GDNF on NMJ	2D	PDMS	Skeletal myocytes (murine) Neurons (murine)	Immunocytochemistry Calcium live imaging	-	GDNF Oxidative stress BTX	Spatially distinct effects of GDNF on MN.	[242]
Organophosphate toxicity	3D	Organoplate - PS	bEnd.3 (murine) C8-D1A (murine) BV-2 (murine)	Immunocytochemistry Acetylcholinesterase assay	1.5 μL h^−1^	DMMP DEMP DECP DCP	OPs penetrate the blood brain barrier (BBB) and rapidly inhibit AChE activity. In vitro toxicity was correlated with available in vivo data.	[193]
Combinatorial systems - Co-culture of liver and neurospheres	3D	PDMS	HepaRG (human) Hepatic stellate cells (human) hNT-2	Immunocytochemistry qPCR LDH activity Metabolic activity TUNEL assay	NA	2,5-hexanedione	Successful co-culture of neuro-spheres and liver spheroids for 14 days. Co-culture was more susceptible to 2,5-hexanedione treatment compared to monocultures.	[194]

## Data Availability

Data is available under reasonable email request.
